# Predominant T-cell epitopes of SARS-CoV-2 restricted by multiple prevalent HLA-B and HLA-C allotypes in Northeast Asia

**DOI:** 10.3389/fimmu.2025.1545510

**Published:** 2025-05-21

**Authors:** Yu Zhao, Min Peng, Chengtao He, Min Li, Xuelian Han, Qiang Fu, Yandan Wu, Fangping Yue, Chunguang Yan, Guangyu Zhao, Chuanlai Shen

**Affiliations:** ^1^ Department of Microbiology and Immunology, Medical School of Southeast University, Nanjing, China; ^2^ Blood Group Reference Laboratory, Nanjing Red Cross Blood Center, Nanjing, China; ^3^ State Key Laboratory of Pathogen and Biosecurity, Academy of Military Medical Sciences, Beijing, China; ^4^ Laboratory of Advanced Biotechnology, Academy of Military Medical Sciences, Beijing, China

**Keywords:** SARS-CoV-2, HLA-B allotype, HLA-C allotype, T-cell epitope, vaccine

## Abstract

Since the outbreak of novel coronavirus pneumonia (COVID-19), numerous T-cell epitopes in severe acute respiratory syndrome coronavirus 2 (SARS-CoV-2) proteome have been reported. However, most of the identified CD8^+^ T-cell epitopes have been restricted primarily by HLA-A allotypes. The epitopes restricted by HLA-B and HLA-C allotypes are limited. This study focuses on the screening of T-cell epitopes restricted by 13 prevalent HLA-B and 13 prevalent HLA-C allotypes, which cover over 70% and 90% of the Chinese and Northeast Asian populations, respectively. Totally, 67 HLA-B restricted and 53 HLA-C restricted epitopes were validated as immunogenic epitopes with a herd predominance rate by peptide-PBMCs *ex vivo* coculture experiments using the PBMCs from convalescent Chinese cohort. In addition, 26 transfected cell lines expressing indicated HLA-B or HLA-C allotype were established, and used in the competitive peptide binding assays to define the affinities and cross-restriction of each validated epitope binding to HLA-B or HLA-C allotypes. These data will facilitate the design of T-cell-directed vaccines and SARS-CoV-2-specific T-cell detection tools tailored to the Northeast Asian population. The herd test of functionally validated T-cell epitopes, and the competitive peptide binding assay onto cell line array expressing prevalent HLA allotypes may serve as an additional criterion for selecting T-cell epitopes used in vaccine.

## Introduction

During the coronavirus disease 2019 (COVID-19) pandemic, extensive research has highlighted the significant role of T cells in combating severe acute respiratory syndrome coronavirus 2 (SARS-CoV-2) infections (1, 2). Current vaccines mostly target the spike protein or the RBD region of the spike protein, aiming to generate neutralizing antibodies that prevent viral entry into host cells. However, the rapid decline of neutralizing antibodies (3–5) and constant emergence of viral variants have significantly reduced the effectiveness of current vaccines (6–10). The widely administered vaccines include inactivated virus vaccines (CoronaVac and BBIBP-CorV), recombinant protein vaccines (NVX-CoV2373), adenovirus vector vaccines (ChAdOx1nCoV-19 and Ad26.COV2.S), and mRNA vaccines (BNT162b2 and mRNA-1273) (11, 12). Among these, only adenovirus vector and mRNA vaccines can elicit efficient T-cell immunity compared to other forms (13, 14). Enormous studies have further demonstrated that mRNA vaccines offer the highest and most stable protection rates (11, 15), which indicates the necessity for designing an ideal vaccine to induce both humoral and cellular immunity, thereby providing a more robust antiviral response. However, the T-cell epitopes derived from mRNA or adenovirus vector vaccines are limited to the spike protein, an area highly susceptible to mutations; this may reduce protective efficacy against viral variants. Multipeptide vaccines based on T-cell epitopes are powerful tools to elicit cellular immunity and generate long-lasting memory T cells. However, due to the herd polymorphism of HLA and the limited number of peptides encompassed in a multipeptide vaccine, it is essential to select epitope peptides that possess strong immunogenicity and a large herd coverage. For the Northeast Asian population, the broad-spectrum and predominant T-cell epitopes of SARS-CoV-2 remain limited.

In our previous work, a repertoire consisting of 120 CD8^+^ T-cell epitopes restricted by 13 predominant HLA-A allotypes in China and Northeast Asia has been defined by cellular functional experiments (16). This study focuses on the screening of T-cell epitopes restricted by 13 predominant HLA-B and 13 prevalent HLA-C allotypes, which cover over 70% and 90% of the Chinese and Northeast Asian populations, respectively. Candidate epitopes were selected by *in silico* prediction and then cocultured *ex vivo* with the peripheral blood mononuclear cells (PBMCs) from convalescent Chinese cohort to identify the immunogenic epitopes, which activate epitope-specific memory CD8^+^ T cells in PBMCs. Finally, 67 HLA-B restricted and 53 HLA-C restricted epitopes were validated with a herd predominance rate. Among the 67 HLA-B restricted epitopes, 26, 36, and 5 ones had herd positive rate of 20%–36.7%, 10%–19%, and <10%, respectively. Of the 53 HLA-C restricted epitopes, 15, 27, and 11 ones had herd positive rate of 20%–27%, 10%–19%, and <10%, respectively. Furthermore, the binding affinities of each validated epitope to HLA-B or C allotypes were assessed by using an array of transfected cell lines expressing indicated HLA-B or HLA-C molecule and competitive peptide binding assay.

## Materials and methods

### Ethical approval, PBMC preparation, and HLA genotyping

The blood samples of convalescents after SARS-CoV-2 infection were collected in the Blood Component Preparation Section of Nanjing Red Cross Blood Center in the form of white blood cell filter trays after red blood cell filtering from March 2022 to March 2024 (the late stage of SARS-CoV-2 outbreak). These blood donors were SARS-CoV-2 negative as confirmed by PCR test upon blood donation but have been defined infection history of SARS-CoV-2. For each donor, a written informed consent was obtained upon blood donation at Nanjing Red Cross Blood Center and informed that their blood samples would be used for patient treatment and scientific research. The collection and use of human blood samples were approved by the Clinical Ethics Committee of Nanjing Red Cross Blood Center (ref: 2022-015-01).

PBMCs were freshly isolated from the white blood cell filter tray and then immediately separated using Ficoll–Paque density-gradient centrifugation. Fresh PBMCs were either used directly or cryopreserved at −80°C for further testing. Genomic DNA was extracted from each PBMCs sample using the Blood DNA Extraction kit from Tiangen Biotech Co., Ltd (Beijing, China) and followed by PCR-sequencing-based typing on HLA-B or HLA-C loci (exon 2 and 3) using the primers recommended by the International HLA working group (IHWG). Sequence alignment was carried out using the SOAPTying software.

### *In silico* prediction of T-cell epitopes and peptide synthesis

T-cell epitopes spanning the spike, envelope, membrane, nucleocapsid, and RNA-dependent RNA polymerase (S, E, M, N, RdRP) proteins of SARS-CoV-2 (Wuhan strain) and presenting different HLA-B or HLA-C allotypes were predicted using five online epitope prediction tools (NetMHCpan 4.1 EL, NetMHCpan 4.1 BA, SMMPMSEC, Consensus, and SYFPEITHI). For each HLA-B or HLA-C allotype and each protein, up to 20 peptides (9 or 10 amino acids/peptide) presenting high scores (highest affinity) in at least two prediction tools were selected as candidate epitopes for further validation. The peptides of candidate epitopes were synthesized by Nanjing GenScript Biotech Co., Ltd. (Nanjing, China) and exhibited a purity >95% as confirmed by HPLC and mass spectrometry analysis. The lyophilized peptides were reconstituted in a DMSO-PBS solution to prepare stock solutions at a concentration of 2 mg/mL for subsequent cellular functional experiments.

### Cell lines and culture

The 293T and HMy2.CIR cell lines were cultured in DMEM and RPMI1640 medium containing 10% fetal calf serum (FBS) and 1% penicillin/streptomycin, respectively. HMy2.CIR cells expressing indicated HLA-B or HLA-C molecule were cultured in RPMI1640 medium containing 10% fetal calf serum at the presence of low concentration of puromycin (1 μg/mL). All cell lines were identified by single nucleotide polymorphism (SNP) testing and incubated at 37°C in an incubator with 5% CO_2_.

### Generation of HMy2.CIR cell lines expressing the indicated HLA-B or HLA-C molecule

The lentiviral vectors pLenti-EF1a-Puro-T2A-GFP expressing the indicated HLA-B or HLA-C molecules were generated in Sangon Biotech Co., Ltd (Shanghai, China) and then were transfected into 293T cells, along with packaging plasmids (pMD2.g and PAX2), using Lipofectamine 3000 (Thermo Fisher Scientific, Waltham, MA, USA). Lentiviral particles were concentrated through ultracentrifugation and stored at −80°C. All plasmids were stored and verified in the Department of Microbiology and Immunology at Medical School of Southeast University.

HMy2.CIR cell line was purchased (Zhongqiao Xinzhou Biotech, Shanghai, China) and maintained in complete IMDM medium with 10% FCS and 1% penicillin/streptomycin in T25 flasks for 48 h, after which the medium was replaced with fresh medium containing lentiviral particles and polybrene (8 µg/mL, Obio Technology, Shanghai, China) for another 24-h culture. Following this, the CIR cells stably expressing HLA-B or HLA-C molecules were selected using puromycin (2 µg/mL, Sigma-Aldrich, St. Louis, MO, USA) and were then stained with APC-conjugated anti-human HLA-A/B/C antibodies (W6/32 clone, BioLegend, San Diego, CA, USA) followed by flow cytometry using Guava^®^ easyCyte™ HT (Luminex Corporation, Austin, TX). The CIR cells transduced with exogenous HLA-B or HLA-C molecular exhibited a significantly higher mean fluorescence intensity (MFI) compared to those transduced with the empty vector.

### Peptide-PBMCs coculture experiment using convalescents’ PBMCs and intracellular IFN-γ staining

Fresh PBMCs (5×10^5^) from each convalescent individual were stimulated with single candidate epitope peptide (20 μg/mL) or without peptide (negative control) for 1 h. Subsequently, a mixture of Brefeldin A and Monensin (BioLegend) was added to the cells for an additional 6-h coculture. After that, the cells were harvested, washed, and blocked with human Fc receptor-blocking reagent (Miltenyi Biotec) for 20 min at 4°C. Then, surface markers were stained with antibodies including FITC-conjugated anti-human CD3 (UCHT1 clone, BioLegend) and APC-conjugated anti-human CD8 (SK1 clone, BioLegend) at pre-titrated concentrations for 30 min at 4°C. After washing, the cells were fixed, permeabilized using Fix&Perm kit (Multi Sciences Co., Ltd, Shanghai, China), and then incubated with PE-conjugated anti-human IFN-γ antibody (B27 clone, BD Bioscience, San Jose, CA, USA) for another 30 min at 4°C. After washing, the cells were harvested and analyzed by flow cytometry (Guava^®^ easyCyte™ HT, Luminex Corporation) to determine the frequencies of IFN-γ^+^ cells in CD3^+^/CD8^+^ populations.

### Competitive peptide binding assay using transfected HMy2.CIR cell lines

The competitive peptide binding assay was used to evaluate the affinity of epitope peptide binding to HLA-B or HLA-C molecule as described in detail in our previous work (16). Briefly, the HMy2.CIR cells constantly expressing indicated HLA-B or HLA-C molecule were incubated with Cy5-labeled reference peptide (defined in published papers or in house) and peptide to be tested. In parallel, the max control well (CIR cells and Cy5-reference peptide) and back group well (CIR cells alone) were performed. After a defined incubation period, the unbound peptides were removed, and the relative binding affinity of the test peptide to the HLA molecule was quantified by the declined MFI of reference peptide binding to the CIR cell surface. The Cy5-labeled reference peptides for each HLA-B or HLA-C molecule are listed in following **Table 1**. The relative binding affinity for given peptide were calculated by the formula: competitive binding (%) = [1 − (MFI_sample_ − MFI_background_)/(MFI_max_ − MFI_background_)] × 100%. The IC50 is the concentration of unlabeled competitor peptide required to inhibit the binding of the Cy5-labeled reference peptide by 50%, which was calculated from the competitive binding inhibition (%) of the sample at 5 and 15 μM. The binding affinity of each unlabeled peptide with the indicated HLA-B or HLA-C molecule was assessed according to the IC50 value. IC50 < 5 μM (5 μM inhibition >50%) means high binding affinity, 5 μM< IC50 < 15 μM (5 μM inhibition <50% but 15 μM inhibition > 50%) means intermediate binding affinity, IC50 > 15 μM means low or no binding affinity (5 μM inhibition 20%–50% or 15 μM inhibition 30%–50% means low binding affinity; 5 μM inhibition < 20% or 15 μM inhibition < 30% means no binding affinity).

**Table 1:** The Cy5-labeled reference peptides for each HLA-B or HLA-C allotype.

Reference peptides	HLA-B or HLA-C allotypes	Resource
Cy5-SLRGLPVCAF	HLA-B4601, B1301	In house
Cy5-MENTTSGFL	HLA-B5401	In house
Cy5-VSYVNVNMGL	HLA-B5801, B4801, C0102, C0401, C0702, C0801, C1402	In house
Cy5-MMWYWGPSL	HLA-B1502, B1302, B0702, B5201, C0303, C1202	In house
Cy5-SASSASSCL	HLA-B3501, C0304, C0602	In house
Cy5-LSAMSTTDL	HLA-C1203	In house
Cy5-LPLLPIFFCL	HLA-B4001, B5101, B1501	In house
Cy5-KRQDILDLWVY	HLA-C0701	(17)
Cy5-LLHERLDEF	HLA-C0302	(18)
Cy5-QQNWWTLLV	HLA-C1502	(19)
Cy5-CYMEAVAL	HLA-B4403	(20)

## Results

### A total of 272 HLA-B restricted and 407 HLA-C restricted CD8^+^ T-cell epitopes were selected as candidate epitopes by *in silico* prediction

A bunch of 9- or 10-mer epitopes restricted by 13 prevalent HLA-B allotypes (HLA-B4601, B4001, B5801, B1502, B5101, B1301, B1302, B1501, B3501, B4403, B5201, BB4801, and B0702, with a gene frequency of >1% for each allotype and a total gene frequency of >70% in Northeast Asia) and 13 prevalent HLA-C allotypes (HLA-C0102, C0602, C0702, C0801, C0304, C0302, C0303, C0401, C1402, C1202, C1502, C1203, and C0701, with a gene frequency of >1% for each allotype and a total gene frequency of >90% in Northeast Asia) were predicted using five advanced epitope prediction tools, following a series of epitope screening principles as previously described (16). Based on the length of each protein and the gene frequency of each HLA-B or HLA-C allotype, 272 HLA-B restricted and 407 HLA-C restricted epitopes as predicted were finally selected as candidate epitopes, of which 171 HLA-B restricted epitopes and 316 HLA-C restricted epitopes were cross-restricted by several HLA-B allotypes and HLA-C allotypes, respectively. Since the cross-restricted candidate epitopes do not need to be synthesized repeatedly, only 101 HLA-B restricted epitopes and 91 HLA-C restricted epitopes were synthesized as peptides for further validation. Among these, 32, 4, 17, 22, and 26 HLA-B restricted epitopes (**Supplementary Figure 1A**, **Supplementary Table S1**) and 29, 5, 16, 18, and 23 HLA-C restricted epitopes (**Supplementary Figure 1B**, **Supplementary Table S2**) harbor in S, E, M, N, and RdRp proteins, respectively.

### Totally, 67 HLA-B restricted and 53 HLA-C restricted epitopes were validated as immunogenic epitopes by cellular functional experiments

To define the immunogenicity of these candidate epitopes, peptide-PBMCs *ex vivo* coculture experiment followed by intracellular IFN-γ staining was used to detect whether the PBMCs from convalescent individuals contained the epitope-specific memory CD8^+^ T cells. When the frequency of IFN-γ^+^/CD8^+^ T cells in the coculture well was twofold greater than those in the negative control well (PBMCs alone), the peptide cocultured with PBMCs was defined as real-world immunogenic epitope, implying the activation of peptide-specific memory CD8^+^ T-cell clones in convalescent peripheral blood.

**Figure 1:**
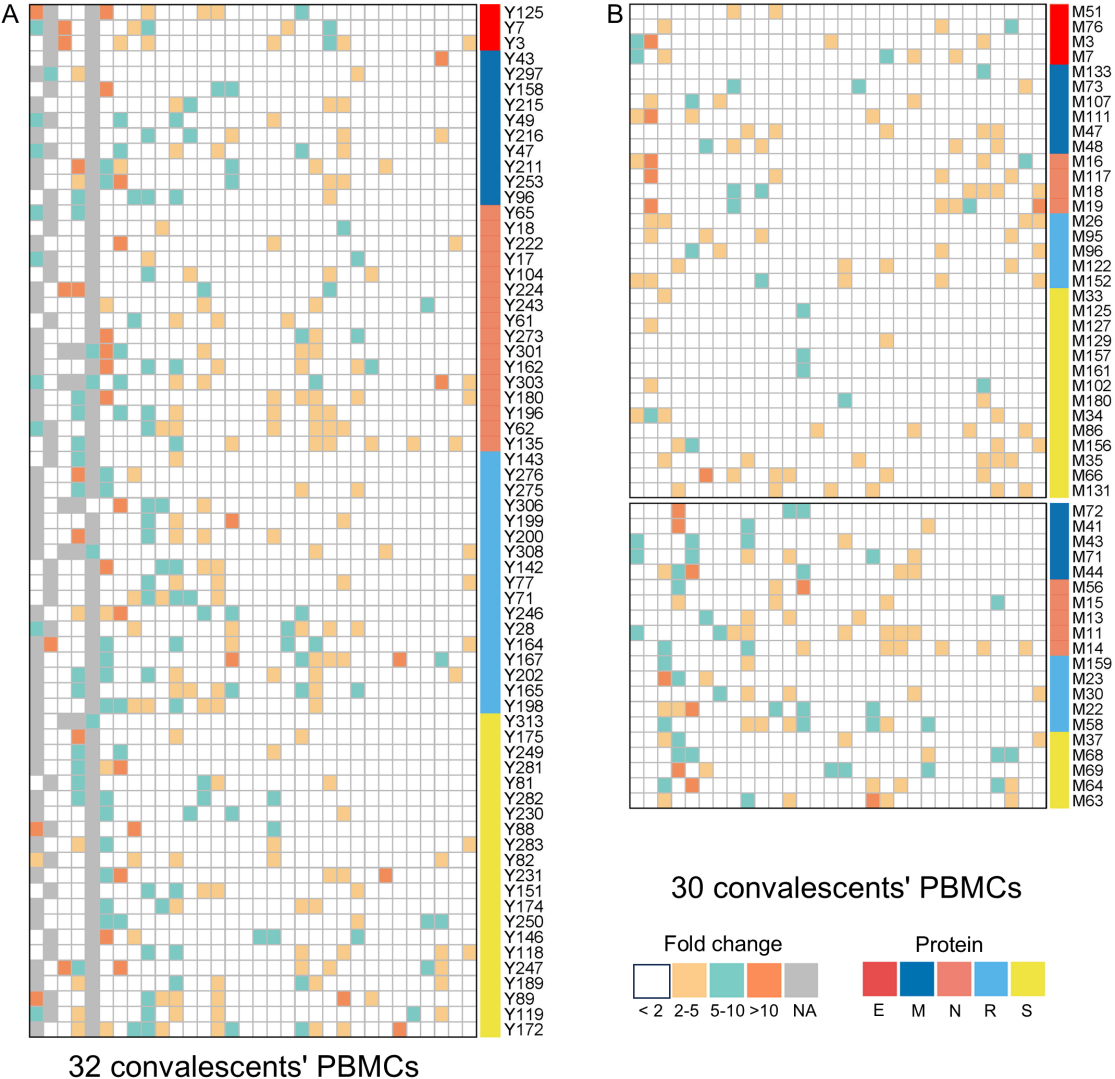
The epitopes inducing CD8^+^ T-cell activation in peptides-PBMCs *ex vivo* cocultures. The heatmap illustrates the results of peptide-PBMCs coculture experiments. Each column represents a convalescent PBMCs sample, and each row represents an indicated epitope peptide. Each color element within the heatmap denote the fold changes of IFN-γ^+^/CD8^+^ T-cell frequency in the peptide-PBMCs coculture relative to its negative control well (PBMCs alone) (white: < 2; yellow: 2–5; cyan: 5–10; orange: >10). Elements in gray indicate no coculture experiment. **(A)** The cocultures of HLA-B restricted epitopes with convalescents’ PBMCs samples. **(B)** The cocultures of HLA-C restricted epitopes with convalescents’ PBMCs samples.

**Figure 2:**
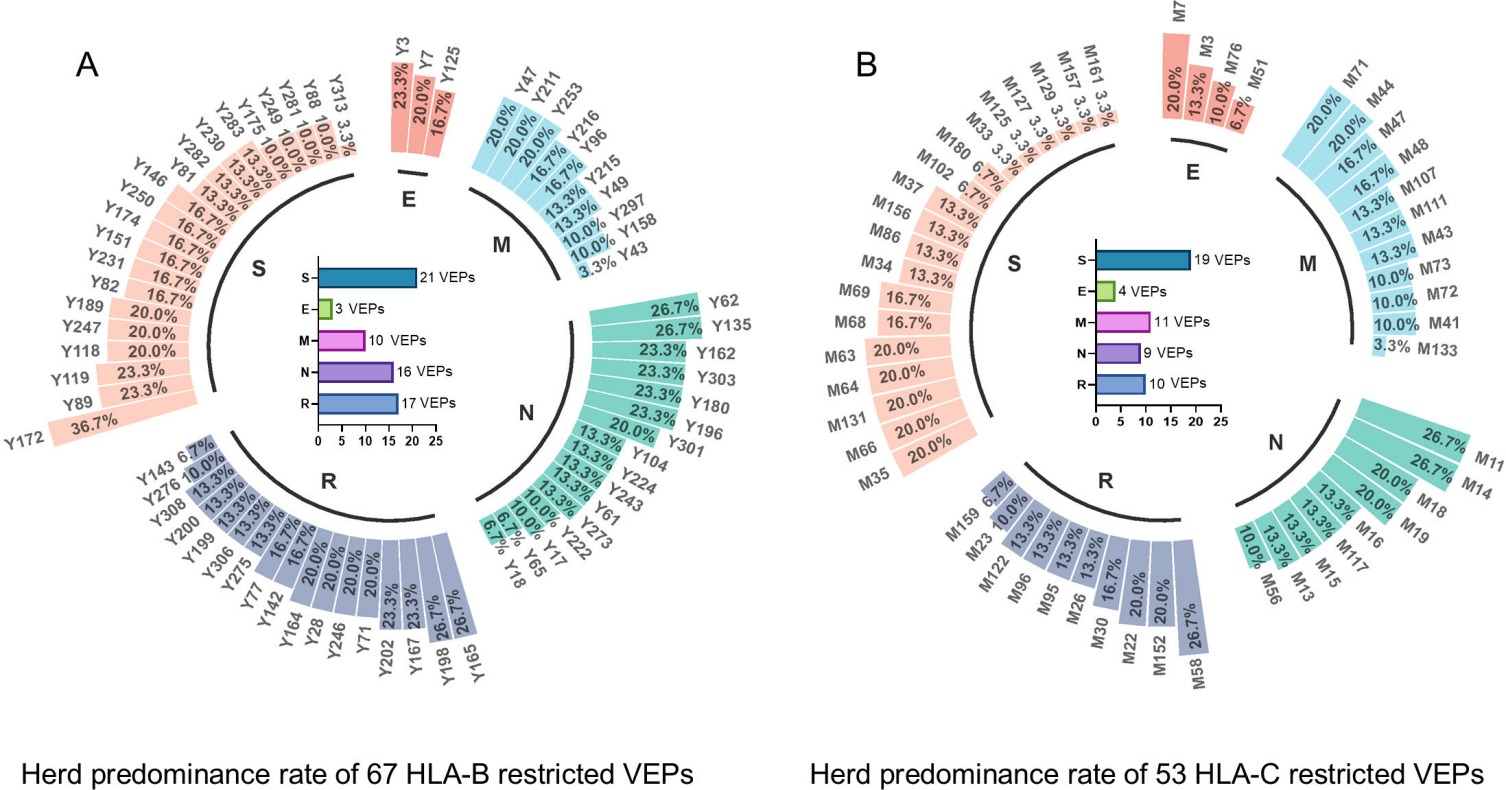
Herd predominance rate of 67 HLA-B restricted VEPs and 53 HLA-C restricted VEPs. Circular histogram displayed the positive rate of each VEP inducing CD8^+^ T-cell activation in the cocultures with 29–32 random convalescents’ PBMCs. **(A)** positive rate of each HLA-B restricted VEP as tested using 32 convalescent samples. **(B)** Positive rate of each HLA-C restricted VEPs as tested using 30 convalescent samples. Internal histogram exhibited the numbers of VEPs derived from different proteins.

**Figure 3:**
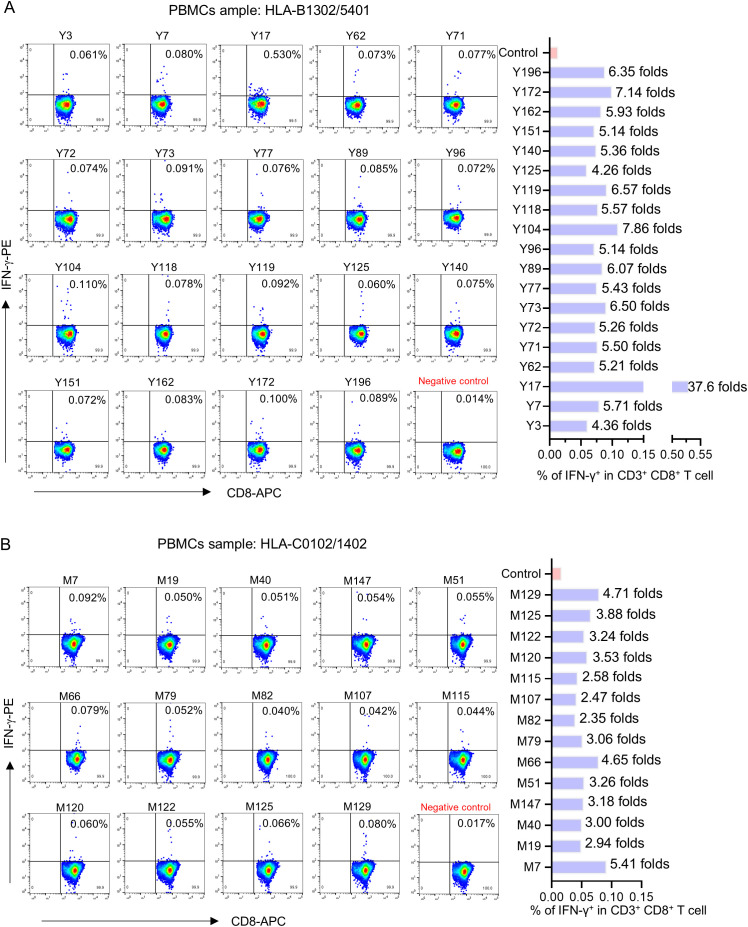
The flow cytometric dot plots of intracellular IFN-γ staining in representative peptide-PBMCs cocultures. After *ex vivo* coculture of PBMCs with candidate epitope peptide, the cells were harvested and followed by intracellular IFN-γ staining using FITC-conjugated anti-human CD3, APC-conjugated anti-human CD8, and PE-conjugated anti-human IFN-γ antibodies. After washing, the cells were harvested and analyzed by flow cytometry to determine the frequencies of IFN-γ^+^ cells in CD3^+^/CD8^+^ populations. Negative control means PBMCs alone well. **(A, B)** Representative flow cytometry plots (left) and the histogram showed the frequency of IFN-γ^+^/CD3^+^/CD8^+^ T cell elicited by each epitope (right).

Totally, each candidate epitope was cocultured with the PBMCs from 29 to 32 convalescents. For 101 HLA-B restricted candidate epitopes and 91 HLA-C restricted candidate epitopes, 32 and 30 convalescents’ PBMCs displayed positive CD8^+^ T-cell responses in the coculture experiments, respectively. Finally, 67 HLA-B restricted candidate epitopes and 53 HLA-C restricted candidate epitopes were validated as immunogenic epitopes, and each validated epitope (VEP) elicited CD8^+^ T-cell activation in one, two, or more convalescents’ samples (**Tables 2**, **3**). The fold changes in IFN-γ^+^/CD8^+^ T-cell frequency in each peptide-PBMCs coculture comparing to its negative control well (PBMCs alone) are presented in **Figure 1**. The positive rate of each VEP inducing CD8^+^ T-cell activation in 29–32 random convalescents’ PBMCs is displayed in **Figure 2**. Most of the in-house validated CD8^+^ T-cell epitopes have a high herd predominance rate in Chinese cohort (3%–36.7%). Of the 67 HLA-B restricted VEPs, 26, 36, and 5 presented 20%–36.7%, 10%–19%, and <10% of herd positive rate, respectively. Of the 53 HLA-C restricted VEPs, 15, 27, and 11 presented 20%–27%, 10%–19%, and <10% of herd positive rate, respectively. In addition, the numbers of HLA-B restricted VEPs derived from S, E, M, N, and RdRp were 21, 3, 10, 16, and 17, respectively, while 19, 4, 11, 9, and 10 for HLA-C restricted VEPs (**Figure 2**). The flow cytometric dot plots of intracellular IFN-γ staining in representative peptide-PBMCs cocultures are displayed in **Figure 3**. The IFN-γ intracellular staining data for all PBMCs samples are presented in **Supplementary Figures S2**, **S3**.

**Table 2:** HLA-B cross-restriction of 67 CD8^+^ T-cell epitopes validated by peptide-PBMCs coculture experiments.

Epitopes	HLA-B allotypes of convalescents displaying positive CD8^+^ T cell response in peptide-PBMCs cocultures					HLA-B allotypes binding to epitopes in competitive peptide binding assay				HLA-B allotypes binding to epitopes as *in silico* predicted
						High	Inter	Low	No	
Y313	5101/5201						5201	5101		5801
Y43	1301/5102						1301			0702, 1302, 5101
Y65	3501/5801	1301/1501				1301	5801, 1501	3501		1301, 4001, 4403
Y143	1301/1501	3501/5801					1301, 1501	3501	5801	1501, 4601, 4801, 5201
Y18	1501/4001	4601/4403					1501	4601	4001, 4403	0702, 5101
Y175	1301/1501	3501/5801	5801/1502			5801	1502	1301, 1501	3501	1502
Y222	1302/1801	1501/5401	1501/5701				1501	1302		4403
Y249	1301/1501	1302/1801	1501/5401				1501	1301, 1302		4601
Y276	1301/1501	1501/1502	3501/5101			1301	5101	1501, 1502	3501	5101, 5201
Y281	1301/1501	1501/1502	1302/1801				1501	1301, 1302	1502	5101
Y297	1501/4001	1301/1501	1501/3503			1501		4001	1301	5201
Y17	3501/5801	1302/5401	1501/3503			3501		1302, 1501	5801	0702, 3501, 5101
Y158	1501/1502	2704/3801	3501/5801			1501	1502, 3501	5801		1501, 1502, 3501, 4601, 5801
Y81	1301/1501	3801/4801	2704/3801	1301/4601		1301		1501, 4601		1301, 1501, 4801
Y104	1302/5401	1301/4001	1301/4601	4601/5701		1302, 4001	1301			1302, 4001, 4801, 5201
Y224	3501/5701	1301/1501	3801/4801	1301/4601			4801	3501, 4601	1301, 1501	4403
Y243	1501/1502	3801/4801	0702/4601	1301/3501		4601	702	1502, 3501	1501, 4801 1301	4601, 5201, 5801
Y275	1301/1501	1501/1502	5801/1502	1501/3503			1501, 1502	1301, 5801		5101, 5201
Y282	1301/1501	1501/1502	3801/4801	1501/5401			1501	1301	1502, 4801	5101
Y306	1302/1801	1302/5401	1501/4001	3801/4801		1302		1501, 4001	4801	5801
Y61	3501/5101	3501/5801	2704/3801	4601/5201		3501	5101, 5801		4601	1301, 1302, 1501, 1502, 4001, 4801, 5201
Y199	1302/5401	3501/5801	3501/5801	4601/4403		3501	1302, 5801	4601		4001
Y200	1301/1501	1302/5401	3501/5801	3801/4801				1301, 1501, 3501, 5801	1302 4801	4001
Y215	3501/5801	1301/4001	1301/4601	4601/4403			1301, 4001, 4601	3501, 5801		4001
Y230	1501/1502	1301/4001	3501/5801	4601/4403		4403	5801	1501, 4001 3501, 4601	1502	4403
Y273	1501/1502	5801/1502	0702/4601	1501/3503			1502	1501, 5801	4601	5101
Y308	5101/5201	0702/4601	1511/4001	0702/4601		0702	4601	5201, 4001 4601	5101	5801
Y49	3501/5801	1302/1801	3501/5801	1501/5401		3501	5801	1302, 1501		0702, 1501, 1502, 3501, 4601, 5101, 5201
Y88	3501/5801	3501/5101	1501/5401				3501	5801, 1501	5101	1301, 1501, 4801, 5201
Y283	1301/1501	1501/1502	1501/5401	1301/1513			1501	1301, 1502		5101, 5201
Y82	3501/5801	3501/5101	3501/5801	1501/5401	1301/5102	3501	5801	1501	5101, 1502	1301, 1302, 4801, 5201
Y216	1302/5401	1301/4001	3501/5801	4601/4403	1301/5102		4001, 5801	1301, 4601	1302, 3501	4001
Y231	1501/1502	1302/1801	1301/4601	4601/4403	1511/4001	4601	1301	1501, 1502, 4001	1302	4403
Y142	1501/1502	1501/4001	3501/5801	3801/4801	2704/3801	5801	1501	1502, 4001	3501, 4801	1501, 4601, 5801
Y151	1302/5401	3501/5801	3801/4801	2704/3801	1501/3503	3501, 1302	3801		5801, 1501	1501, 1502, 3501, 4601
Y174	1501/1502	1501/4001	3501/5801	5801/1502	0702/4601	1502	1501	5801, 4601	4001, 3501	1502
Y250	1501/1502	1302/1801	1501/3503	1301/3501	1301/5102			1302, 3501	1501, 1502	4601
Y77	1302/5401	3501/5801	2704/3801	4601/4403	1301/1513	1302	5801, 4601	4403	3501, 4403, 1301	1301, 1302, 5101, 5201, 5801
Y125	1501/1502	1302/5401	3801/4801	2704/3801	5801/1502	1501	5801	1502	1302, 4801	1501, 1502, 3501, 4403, 4601, 5801
Y146	1501/1502	3501/5101	4601/3501	1501/5401	1501/3503	1501	1502	4501	3501	1501, 4601
Y7	3501/5801	3501/5701	3501/5101	1302/5401	4601/5201		3501, 5801	5801	4601	0702, 1301, 3501, 4001, 4601, 4801, 5101, 5201, 5801
	1301/4601									
Y47	3501/5801	1302/1801	3501/5801	2704/3801	5801/1502	3501, 1502		4601	5801, 1302	0702, 1501, 1502, 3501, 4601
	4601/4403									
Y71	3501/5101	1302/5401	1501/4001	3501/5801	2704/3801		3501, 1501, 4001	5101	1302, 5801	1301, 1302, 4001, 4403
				
Y118	1302/5401	3501/5801	5801/1502	4601/4403	1301/5102	1302	4403, 1302	3501, 4601, 5102	5801, 1502	1302, 5201
	1301/1513									
Y246	1301/1501	1501/1502	1302/1801	3801/4801	3501/5801		3801	1502 4801 3501 5801	1301, 1501, 1302	4601, 5101
	5801/1502									
Y28	3501/5101	3501/5801	4601/5201	5801/1502	1301/4601	4601, 1501	3501	1502, 1301	5101, 5801	0702, 1501, 4601
	1501/3503									
Y247	1301/1501	1302/1801	5801/1502	1301/4601	1301/3501	5801	1302 4601	1301, 1501	1502, 3501	4601, 5801
	1301/5102									
Y301	5101/5201	1501/1502	1302/1801	3801/4801	5801/1502	5801	0702, 4601	5101, 1501, 1502	1302, 480,1 5201	5801
	0702/4601									
Y164	1501/4001	1501/1502	3501/5801	3501/5801	0702/4601		1501, 1502, 5801	3501, 0702	4001, 4601, 1301	1502
	1301/1513									
Y189	1301/1501	3501/5801	2704/3801	5801/1502	0702/4601	4601, 0702	1301, 3501	5801	1501, 1502	3501, 4601, 5801
	1301/5102									
Y211	1301/1501	1501/1502	1302/1801	3501/5801	0702/4601		1501, 4001	1302, 3501, 4601	1301, 1502, 5801, 0702	4001, 4403
	1511/4001									
Y253	1301/1501	1501/1502	1302/1801	3501/5801	1301/4601	4601	1301 1302 4403	1501, 5801	1502, 3501	4601, 4801
	4601/4403									
Y89	3501/5801	1302/5401	1501/4001	3501/5801	2704/3801	1302	4001	5801, 5401, 1501, 4403, 4601	3501, 1302	1301, 1302, 5201
	4601/4403	4601/5701								
Y119	3501/5801	1301/1501	1302/5401	3501/5801	2704/3801		1302	3501, 5801, 1301	1501	1302, 4001
	1511/3502	1301/5102								
Y162	1501/1502	1302/5401	3501/5801	2704/3801	5801/1502	4601	1502, 5801	1501, 3501	1302	1502
	1501/3503	4601/5701								
Y167	1501/1502	3501/5801	5801/1502	0702/4601	1301/4601		1502, 3501, 4601	1501, 5801, 0702, 4403, 1301	1301	1502, 3501, 4601
	4601/4403	1301/5102								
Y202	1301/1501	1501/1502	1302/5401	3501/5801	1501/5401		4601	1501, 1502, 5801 0702	1301, 1302 3501	4001
	0702/4601	1501/5701								
Y303	5101/5201	1302/1801	3501/5801	3801/4801	0702/4601	5801	1302	5101, 5201, 3501, 4801, 4601	1302, 0702	5801
	1301/5102	1301/1513								
Y180	1301/1501	1501/1502	1501/5401	5801/1502	0702/4601		1501	1301, 1502, 4601	5801, 0702	3501
	1501/3503	1301/1513								
Y196	1301/1501	1302/1801	1302/5401	3501/5801	1501/5401	4601	1302	1301, 1501, 3501	5801, 0702	4001, 4801
	0702/4601	1301/4601								
Y3	3501/5701	1302/1801	1302/5401	1501/5401	1301/4601	4403	1501	3501, 1302, 1301	1301, 4601	0702, 1502, 4601, 4801, 5101, 5201
	4601/4403	1301/1513								
Y62	3501/5801	1302/5401	1501/4001	3501/5801	1501/5401	3501	5801, 1501, 4601, 4403	1302, 4001	0702	1301, 1302, 1501, 1502, 3501, 4403, 4601, 4801, 5101, 5201, 5801
	0702/4601	1301/4601	4601/4403							
Y96	1301/1501	3501/5101	1302/5401	3501/5801	1301/4601	1301, 5101	3501, 1302	1501, 4601	5801	1301, 1302, 5101, 5201
Y165	1301/1501	3501/5801	1301/4001	2704/3801	3501/5801	3501	1301, 1502, 0702, 4601	1501, 4001	5801	1502, 3501, 4601
	5801/1502	0702/4601	1501/3503							
Y198	1501/1502	1302/1801	3501/5101	1302/5401	3501/5801		1302	1501, 5101, 4801, 4601	1502, 3501 5801	4001, 4403
	3801/4801	2704/3801	0702/4601							
Y135	1301/1501	3501/5801	3501/5801	0702/4601	1301/4601	1501, 4601	5801, 0702	3501	1301	1501, 1502, 4601
	4601/5701	1511/3502	1501/5701							
Y172	1301/1501	1501/1502	3501/5101	1302/5401	1501/4001	1501, 5801	1502, 3501	1302, 4001, 4601, 4403	1301, 5101, 0702	1301, 1302, 1501, 1502, 3501, 4403, 4601, 4801, 5101, 5201, 5801
	2704/3801	3501/5801	5801/1502	0702/4601	4601/4403					

**Table 3:** HLA-C cross-restriction of 53 CD8^+^ T-cell epitopes validated by peptide-PBMCs coculture experiments.

Epitopes	HLA-C allotypes of convalescents displaying positive CD8^+^ T cell response in peptide-PBMCs cocultures					HLA-C allotypes binding to epitopes in competitive peptide binding assay				HLA-C allotypes binding to epitopes as *in silico* predicted
						High	Inter	Low	No	
M33	0302/0304						0302	0304		0102, 0302, 0303, 0304, 0801, 1202, 1203, 1402, 1502
M125	0602/0610						0602			0602, 0701, 0702
M127	0602/0702							0602	0702	0602, 1203
M129	0102/0302								0102, 0302	0602, 0701, 1203
M133	0716/1510									0602, 1202, 1203 0702
M157	0602/0610							0602		0702
M161	0602/0610								0602	0801
M51	0602/1504	0102/0311				0602	0102			0302, 0701, 1202, 1203, 1402
M102	0602/0702	0716/1510					0602	0702		0401
M180	0602/0311	0716/1510						0602		1402
M159	0102/0303	0702/0327				0702	0102, 0303			0801, 1202
M73	0602/1504	0102/0134	0302/0311			0602	0302	0102		0302, 0303, 0304, 0401, 0602, 0801, 1203, 1502
M23	0102/0303	0801/0315	0401/1402			0303	0102	0801	0401	0102, 0302, 0303, 0304, 0602, 0701, 0801, 1202, 1203, 1402, 1502
M72	0801/0315	0702/0401	0302/0304			0801		0702, 0401	0302, 0304	0302, 0602, 0701, 1202, 1203, 1402
M41	0801/0315	0702/0327	0102/0602				0801	0702	0102	0102, 0302, 0303, 0304, 0602, 0701, 0702, 1202, 1203, 1402
M56	0801/0315	0801/0602	0102/1203				0801	0102	0602	0302, 0303, 0304, 0801, 1202, 1502
M3	0102/0401	0602/0702	0702/0611	0716/1510		0102, 0401	0702	0602		0102, 0302, 0303, 0304, 0401, 0801, 1202, 1203, 1402, 1502
M16	0102/0401	0602/0702	0716/1510	0302/0311		0302	0102, 0401	0602	0702	0102, 0302, 0303, 0401, 0602, 0801, 1202, 1203, 1402
M26	0602/0702	0302/0304	0302/0311	0102/0801			0304, 0302	0602, 0702	0102	0102, 0302, 0303, 0304, 0701, 1202, 1203, 1402
M34	0102/0401	0602/0702	0302/0304	1601/0640		0102	0401	0302, 0304		0102, 1402, 1502
M76	0302/0304	0702/0310	0702/1402			0304	0302, 0702		1402	0303, 0304, 0401, 0602, 0701, 0702, 0801, 1202
M86	0401/0202	0102/1402	0102/0123	0302/0311			0102, 0401	1402	0302	0304, 0801
M95	0602/0702	0102/0304	0102/0304	0702/1402			1402, 0102	0602, 0702		0401
M96	1402/0303	0801/0311	0702/0310	0102/0801		0801	1402	0303	0702, 0102	0401, 0801
M107	0602/0702	1402/0303	0801/0311	0102/1402		1402	0303	0801, 0702	0102, 0602	0401, 0702, 1402
M111	0102/0401	0602/0702	1402/0303	0702/1218		0401	0602	0102	0702, 1402	0401, 0602, 0701, 0801, 1202
M117	0602/0702	0102/0311	0702/0310	0702/1402		0702, 0602		0102, 1402		0602, 0701
M122	0304/1202	0602/0311	0102/0302	0702/1402			0702	0602, 0102, 0302	0304	0602, 0702, 1402
M156	0304/1202	1402/0303	1601/0640	0102/0801			0102	0304, 1202, 1402	0303	0702
M15	0801/0315	0801/0602	0602/0302	0702/1543			0801, 0602	0302, 0702		0102, 0302, 0303, 0304, 0602, 0701, 0801, 1202, 1203, 1402, 1502
M13	0401/1402	0702/0327	0702/0401	0304/1402		0304	0401, 0702	1402		0102, 0302, 0303, 0304, 0401, 0801, 1202
M43	0406/0602	0602/0701	0702/0327	0102/0401		0701	0602, 0702	0401, 0102		0102, 0302, 0303, 0304, 0401, 0602, 0701, 0702, 0801, 1202, 1203, 1402, 1502
M37	0102/0303	0801/0315	0102/0401	0602/0801		0303, 0102		0801	0401, 0602	0102, 0302, 0303, 0304, 0801, 1202, 1203, 1502
M47	0102/0702	0102/0311	0102/0302	0716/1510	1601/0640	0102, 0702		0302		0102, 0401, 0702, 1402
M48	0102/0304	0602/1504	0102/0304	0102/0602	1601/0640		0304	0102, 0602		0102, 0303, 0304, 0401, 0801, 1502
M68	0801/0315	0602/0701	0102/0602	0702/1543	1502/0311		1502	0801, 0602, 0701	0702, 0102	0302, 0701, 1202
M30	0102/0602	0702/0327	0702/0401	0702/0311	0602/0801	0602	0102	0702, 0401, 0801		0102, 0602, 0701, 1502
M69	0801/0315	0401/1402	0102/0801	0102/0401	0102/0602			0102, 0801, 1402, 0401	0602	0302, 1202
M7	0102/0401	0602/0702	0302/0304	0102/0302	0102/1402	0102	0602, 0401	0702, 0304, 0302	1402	0102, 0302, 0303, 0304, 0401, 0602, 0701, 0702, 0801, 1202, 1203, 1502
	0102/0602									
M18	0602/1504	0102/0304	0102/0123	0716/1510	1601/0640		0102		0602, 0304, 0801	0102
	0102/0801									
M19	0102/0801	0602/1504	0702/0310	0102/0602	0102/0123	0602		0702, 0102	0801	0102, 0602, 1203
M35	0302/0304	0102/0702	0102/0134	0716/1510	1601/0640	0702	1402	0102	0302, 0304	0102, 0401, 0702, 0801, 1402
	0702/1402									
M66	0102/0304	0602/1504	0102/0311	0304/1203	0102/0302	0304	0602		0102	0302, 0303, 0304, 0801
	0716/1510									
M131	0304/1202	0102/0311	0702/0611	0702/1218	1601/0640		0304, 0102	0702, 1202	0302	0602, 1502
	0302/0311									
M152	0102/0401	0602/0702	0102/0304	0602/0311	0702/0310		0401, 0304	0102, 0801, 0702	0602	0702
	0102/0801									
M71	0406/0602	0602/0701	0702/0327	0702/0401	0801/0302	0302	0702, 0701	0401, 0602, 0801		0302, 1202, 1203
	0702/0311									
M22	0102/0303	0801/0315	0602/0701	0801/0602	0302/0304	0801, 0302	0304	0102, 0303	0701, 0602	0102, 0302, 0303, 0304, 0801, 1202, 1203, 1402
	0801/0302									
M64	0102/0303	0602/0701	0801/0302	0602/0801	0702/1543	1502	0302	0102, 0303, 0801	0702	0302, 0401, 0701, 1202
	1502/0311									
M63	0102/0303	0702/0327	0702/0401	0801/0302	0602/0302	0302	0303	0102, 0702	0401, 0801, 0602	0302, 1202, 1203
	1502/0311									
M44	0102/0303	0801/0315	0602/0701	0302/0304	0702/0311	0102, 0302		0303, 0801, 0701	0602, 0304 0702	0102, 0302, 0303, 0304, 0801, 1202, 1203, 1402, 1502
	0602/0801									
M11	0406/0602	0102/0602	0102/0401	0702/0327	0102/0401	0602	0102, 0702	0401, 0302		0102, 0302, 0303, 0304, 0602, 0701, 0702, 0801, 1202, 1203, 1402, 1502
	0602/0302	0702/0311	0602/0801							
M58	0102/0303	0702/0327	0801/1653	0702/0401	0302/0304	0302	0303	0102, 0702, 0401	0801, 0602	0302, 0303, 0304
	0801/0302	0602/0302	0102/0602							
M14	0102/0303	0702/0327	0302/0304	0602/0302	0602/0801	0303, 0302	0702, 0304	0801, 0102	0602	0102, 0302, 0303, 0304, 0602, 0701, 0702, 0801, 1202, 1203, 1402, 1502
	0303/0303	0801/0323	0602/0311							

### Binding affinity and cross-binding of validated epitopes with corresponding HLA-B and C allotypes were analyzed using competitive peptide binding assay

To evaluate the affinity between VEPs and HLA-B or HLA-C allotypes, 26 transfected HMy2.CIR cell lines stably expressing the indicated HLA-B or C allotype were successfully constructed and identified, including HLA-B4601, B4001, B5801, B1502, B5101, B1301, B1302, B1501, B3501, B4403, B5201, B4801, B0702; HLA-C0102, C0602, C0702, C0801, C0304, C0302, C0303, C0401, C1402, C1202, C1502, C1203, and C0701. HMy2.CIR is a human B lymphocyte strain with HLA class I antigen deficiency, which does not express HLA-A and HLA-B molecules and only expresses trace HLA-Cw4. The CIR cells transduced with exogenous HLA-B or HLA-C gene displayed GFP fluorescence under a fluorescent microscope (**Supplementary Figure S4**) and exhibited a significantly higher fluorescence intensity compared to those transduced with empty vector, after being stained with APC-conjugated anti-HLA-A/B/C antibodies (**Supplementary Figure S5**).

**Figure 4:**
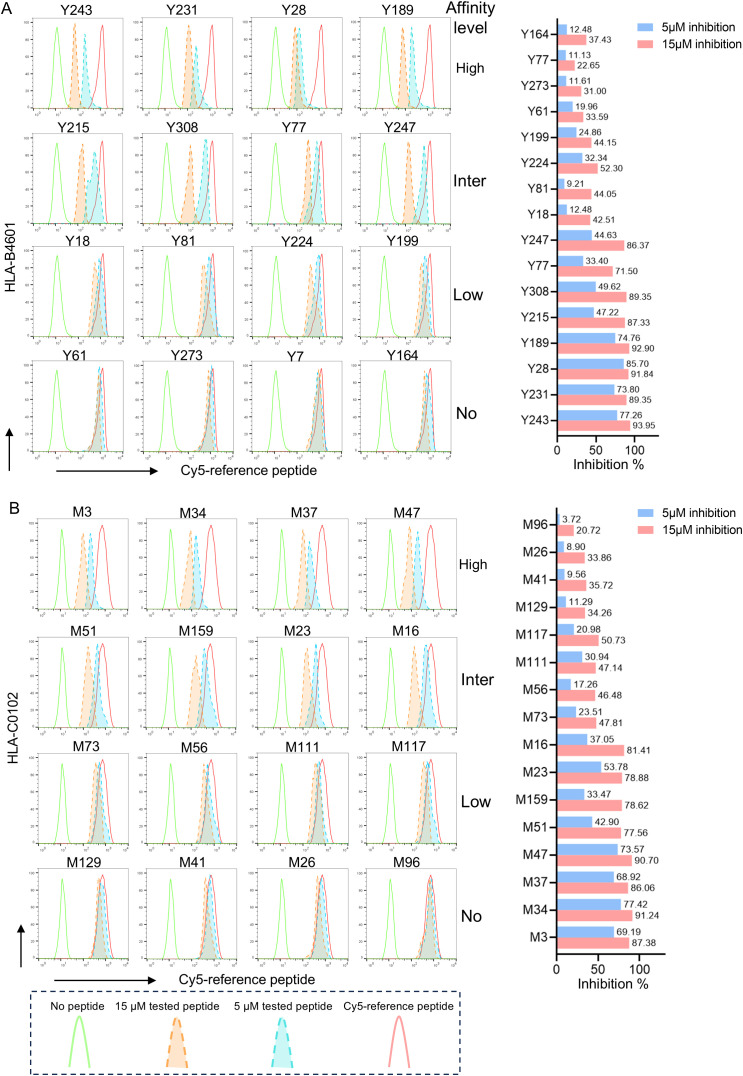
The representative flow cytometric histograms of VEPs binding to HLA-B and HLA-C allotypes in competitive peptide binding assays. The HMy2.CIR cells constantly expressing indicated HLA-B or HLA-C molecule were incubated with Cy5-labeled reference peptide and the no-labeled epitope peptide. After a defined incubation period, the unbound peptides were removed, and the relative binding affinity of the tested epitope peptide to the HLA-B or HLA-C molecule was quantified by the declined MFI of reference peptide binding to the CIR cell surface at different concentration of tested epitope peptide (5 and 15μm). In parallel, the max control well (CIR cells and Cy5-reference peptide; red solid-line peak in flow cytometry histograms) and background well (CIR cells alone; green solid-line peak in flow cytometry histograms) were performed. **(A)** Representative flow cytometry plots reflecting the affinity between epitopes and HLA-B4601 (left), the histogram showed the inhibition rates of each epitope at 5 and 10 μM concentrations against the Cy5-reference peptide (right) when incubated with CIR-B4601; **(B)** representative flow cytometry plots reflecting the affinity between epitopes and HLA-C0102 (left), the histogram showed the inhibition rates of each epitope at 5 and 10 μM concentrations against the Cy5-reference peptide (right) when incubated with CIR-C0102.

Then, competitive peptide binding assays were performed. Briefly, each HMy2.CIR cell line expressing indicated HLA-B or HLA-C molecule was cocultured with the corresponding Cy5-labeled reference peptide and the no-labeled competitor VEP peptide and followed by flow cytometry analysis. The flow cytometric line diagrams showed that most of the tested VEP peptides could efficiently compete with the Cy5-labeled reference peptide and bind to the HLA-B or HLA-C molecules onto the relative CIR cell lines, leading to leftward shift of the fluorescence peak compared to the max control well (CIR cells and Cy5-reference peptide) (**Figure 4**). **Supplementary Tables S3**, **S4** exhibited the binding affinity of each HLA-B and HLA-C allotype with corresponding VEPs, respectively. Most of the HLA-B and HLA-C allotypes could crossly bind to 3–24 VEPs with high or intermediate affinity, such as HLA-B1501 and HLA-C0102 efficiently bound to 24 and 17 VEPs, respectively. On the other hand, most of VEPs showed cross-binding to several HLA-B or HLA-C molecules with high- or inter-affinity. The HLA restriction of each VEP is further summarized in **Tables 2**, **3** by combining the data of cellular functional assay, competitive peptide binding assay, and *in silico* prediction. These data suggest that most of the in-house validated CD8^+^ T-cell epitopes have a large coverage in China and Northeast Asia. **Supplementary Figures S6**, **S7** displayed the flow cytometric histograms of each VEP binding to multiple HLA-B or HLA-C allotypes in competitive peptide binding assays.

## Discussion

Humoral immunity against SARS-CoV-2 wanes quickly and thus cannot maintain long-term antiviral immunity (3–5) and exhibits weak resistance to viral variants (9, 10). Conversely, T cells against SARS-CoV-2 showed powerful effects and long-lasting memory immunity for defending against infection (21, 22). More importantly, the T-cell epitopes possess a much larger repertoire and much higher conservatism as compared with the B-cell epitopes, implying stronger resistance to viral variants. Therefore, a lot of attention has changed to the multipeptide vaccines based on T-cell epitopes, such as CoVepiT (NCT04885361), EpiVacCorona (NCT04527575) (23), IMP CoVac-1 (containing six T-cell epitopes) (24), PepGNP-SARSCoV2 (NCT04935801, containing nine T-cell epitopes) (25), UB-612 (containing five T-cell epitopes and RBD-Fc) (26), and BNT162B4 (mRNA vaccine based on T-cell epitopes) (27). To design an effective multipeptide vaccine tailored to an indicated geographic population, the T-cell epitopes should be selected according to the following criteria: 1) broad spectrum, the epitopes can bind to various prevalent HLA allotypes with a large coverage of the indicated population; 2) high herd predominance, the epitope has a high positive rate to induce T-cell response in the indicated population; 3) conservation, the epitopes are located in the conserved protein regions and no substitution of anchor amino acids in viral evolutionary history; and 4) high affinity and strong immunogenicity, the epitope can strongly bind to HLA molecule to maintain the peptide–MHC complexes onto T-cell surface for extended duration and robust T-cell response.

Currently, numerous studies have reported at least 1,000 functionally validated CD8^+^ T-cell epitopes, but most ones are mainly restricted by HLA-A allotypes, particularly the common allotypes such as HLA-A2 or A24 (28). Nevertheless, HLA-B and HLA-C allotypes, as classic HLA class I molecules, can also present antigenic peptides to induce robust T-cell responses, so HLA-B and HLA-C restricted epitopes can serve as important supplements of peptide cocktail used in multipeptide vaccines, especially for the individuals carrying rare HLA-A alleles. For example, if the individual possesses rare HLA-A allotype but dominant HLA-B or C allotypes, the HLA-B or HLA-C restricted epitopes also can elicit robust T-cell immunity to defend against viral infection. This strategy, utilizing the cocktail of HLA-A, HLA-B, and HLA-C restricted T-cell epitopes in vaccine, enables a minimal number of peptides to cover a broader population. Moreover, several studies have indicated that certain HLA-B or HLA-C allotype carriers are more susceptible to SARS-CoV-2 infection and exhibited higher rates of severe disease and mortality (29–33). These findings underscore the necessity of considering the HLA-B and HLA-C genotypes of vulnerable populations when designing vaccines.

Different from the screening of T-cell epitopes from other researchers, this study aims to provide a pool of broad spectrum and herd predominant CD8^+^ T-cell epitopes, which are derived from various SARS-CoV-2 proteins, tailored to the polymorphism of HLA-B and HLA-C allotypes in Northeast Asia, and has a high herd predominance rate in Chinese cohort. More importantly, the epitopes were validated by using convalescents’ fresh PBMCs and peptide-PBMCs *ex vivo* cocultures, which are cellular functional experiments to determine the epitope-specific memory CD8^+^ T-cell clones in convalescent peripheral blood. All enrolled convalescents had recovered from SARS-CoV-2 infection at least 2 years prior, which ensured that the T-cell responses elicited by epitopes were from memory T cells. In addition, the HLA restriction of T-cell epitope is difficult to determine because there is no standard method for now. To solve this issue, this study constructed a series of transfected HyM2.CIR cell lines, which constantly express indicated HLA-B or HLA-C molecules, and followed by an abundance of competitive peptide binding assay, and finally defined the affinities and cross-restriction of each validated epitope with associated HLA-B or HLA-C allotypes depending on the relatively objective data.

In summary, this study provided a verified repertoire of T-cell epitopes, which derived from SARS-CoV-2 main proteins and restricted by predominant HLA-B and HLA-C allotypes covering most of China and Northeast Asia population. These data will facilitate the design and development of SARS-CoV-2 peptide vaccines and specific T-cell detection systems for Northeast Asia. The herd test of functionally validated T-cell epitopes and the competitive peptide binding assay onto cell line array expressing prevalent HLA allotypes may serve as an additional criterion for selecting T-cell epitopes used in vaccine.

## Data Availability

The original contributions presented in the study are included in the article/**Supplementary Material**. Further inquiries can be directed to the corresponding authors.
