# Supplementary material for: Predominant T-cell epitopes of SARS-CoV-2 restricted by multiple prevalent HLA-B and HLA-C allotypes in Northeast Asia
Source: Front Immunol. 2025 May 21;16:1545510. doi: 10.3389/fimmu.2025.1545510 (PMC12133982; doi:10.3389/fimmu.2025.1545510)
Supplement: Supplementary file 1 [file DataSheet1.pdf]

Predominant T-cell epitopes of SARS-CoV-2 restricted by multiple prevalent HLA-B and C allotypes in Northeast Asia

Yu Zhao[1†], Min Peng[1†], Chengtao He[2], Min Li[3,4], Xuelian Han[3,4], Qiang Fu[2], Yandan Wu[1], Fangping Yue[1], Chunguang Yan[1], Guangyu Zhao[3,4*], Chuanlai Shen[1*]

[1] Department of Microbiology and Immunology, Medical School of Southeast University, Nanjing, China 210009
[2] Nanjing Red Cross Blood Center, Nanjing, China 210003
[3] State Key Laboratory of Pathogen and Biosecurity, Academy of Military Medical Sciences, Beijing, China 100071
[4] Laboratory of Advanced Biotechnology, Academy of Military Medical Sciences, Beijing, China 100071

[†] These authors contributed equally to this work.

* Correspondence:
Guangyu Zhao*: E-mail: guangyu0525@163.com, ORCID: 0000-0002-0925-5216
Chuanlai Shen*: E-mail: chuanlaishen@seu.edu.cn, ORCID: 0000-0002-3748-3742

This work was funded by the National Key Research and Development Program of China(2022YFC230410)

Supplementary figures

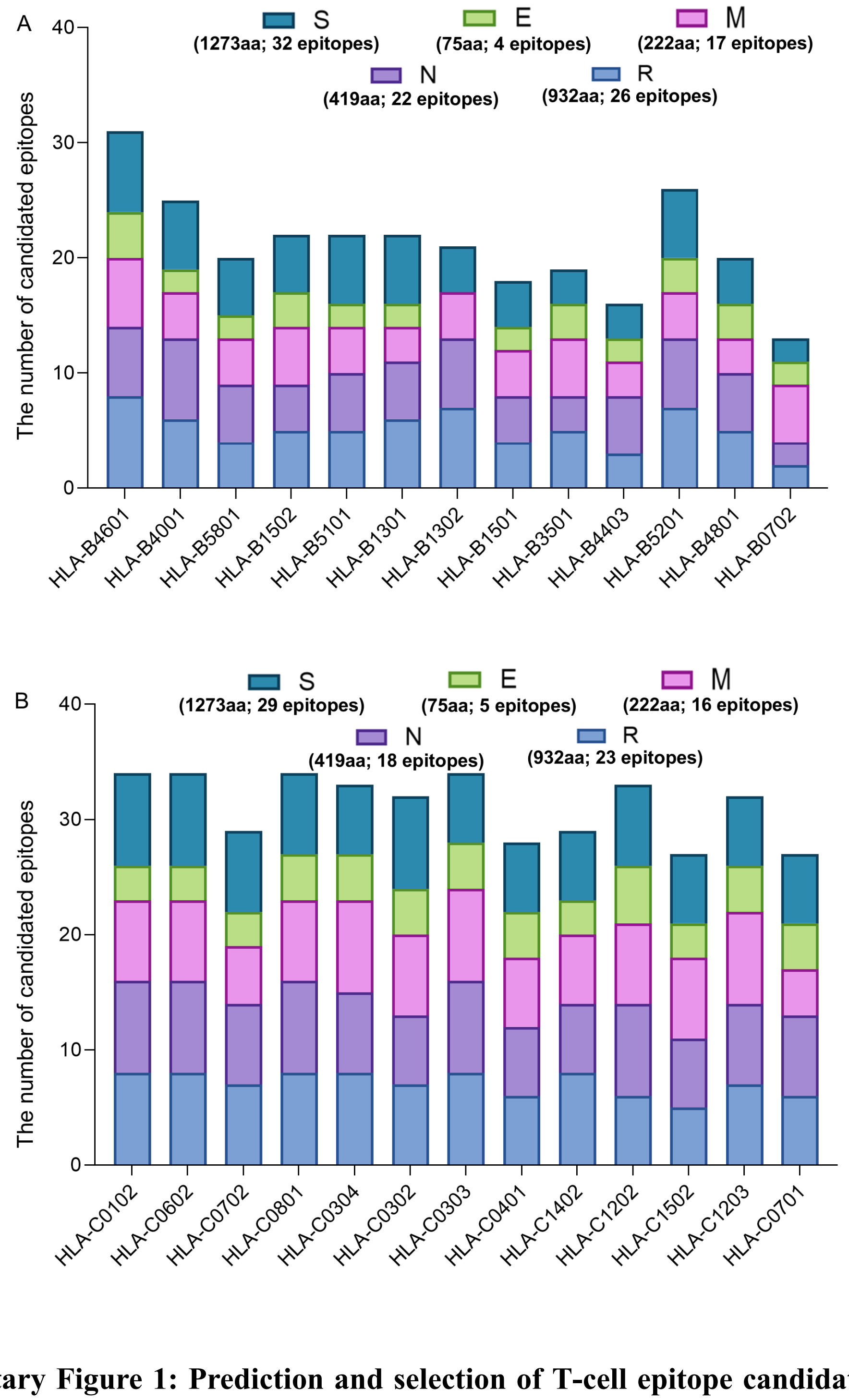

Supplementary Figure 1: Prediction and selection of T-cell epitope candidates derived from SARS-CoV-2 main proteins and restricted by 13 prevalent HLA-B allotypes. The numbers of epitope candidates harboring in each protein and restricted by each HLA-B allotype. (B) The numbers of epitope candidates harboring in each protein and restricted by each HLA-C allotype.

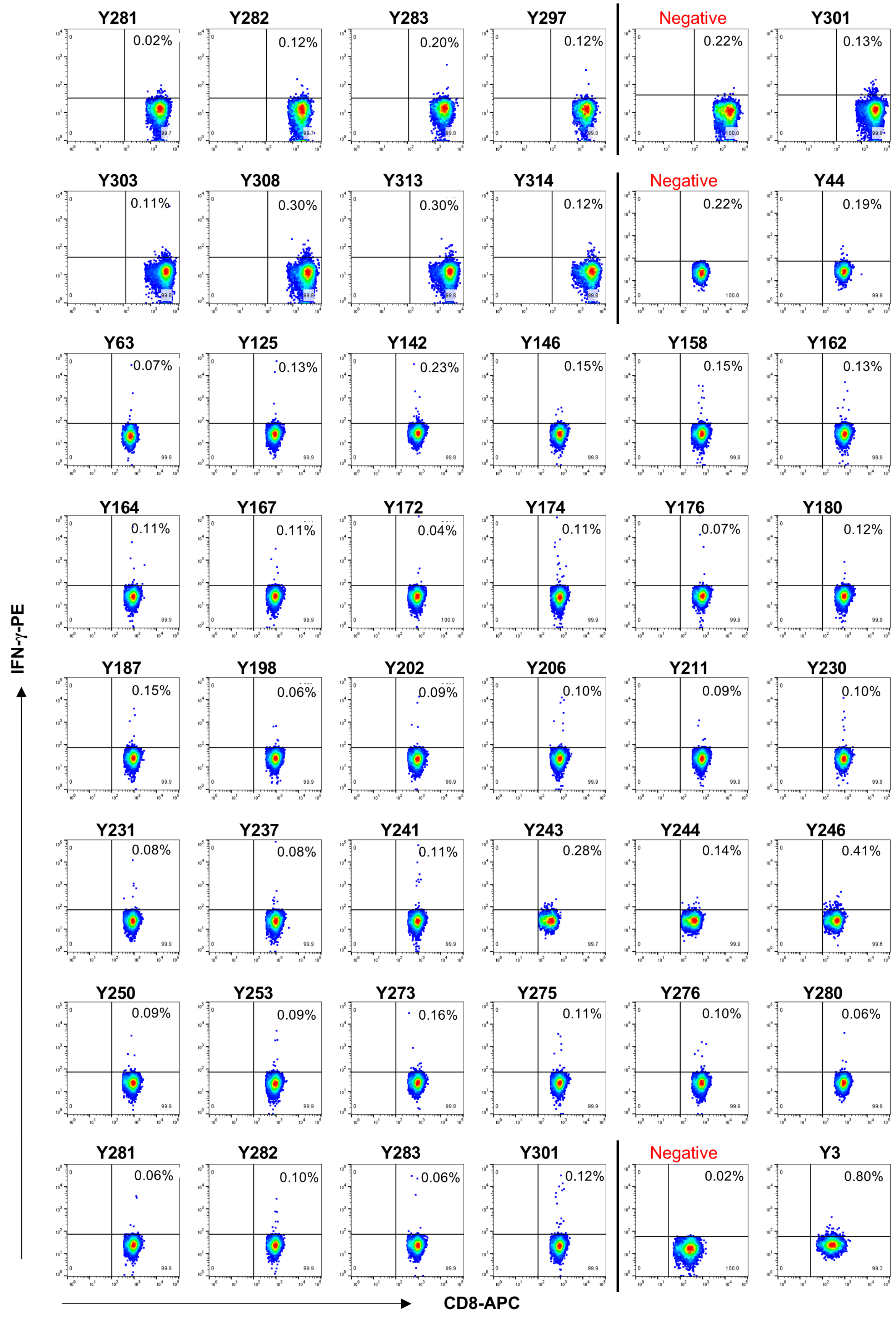
Y281 0.02%
Y282 0.12%
Y283 0.20%
Y297 0.12%
Negative 0.22%
Y301 0.13%
Y303 0.11%
Y308 0.30%
Y313 0.30%
Y314 0.12%
Negative 0.22%
Y44 0.19%
Y63 0.07%
Y125 0.13%
Y142 0.23%
Y146 0.15%
Y158 0.15%
Y162 0.13%
Y164 0.11%
Y167 0.11%
Y172 0.04%
Y174 0.11%
Y176 0.07%
Y180 0.12%
Y187 0.15%
Y198 0.06%
Y202 0.09%
Y206 0.10%
Y211 0.09%
Y230 0.10%
Y231 0.08%
Y237 0.08%
Y241 0.11%
Y243 0.28%
Y244 0.14%
Y246 0.41%
Y250 0.09%
Y253 0.09%
Y273 0.16%
Y275 0.11%
Y276 0.10%
Y280 0.06%
Y281 0.06%
Y282 0.10%
Y283 0.06%
Y301 0.12%
Negative 0.02%
Y3 0.80%
IFN-γ-PE
CD8-APC

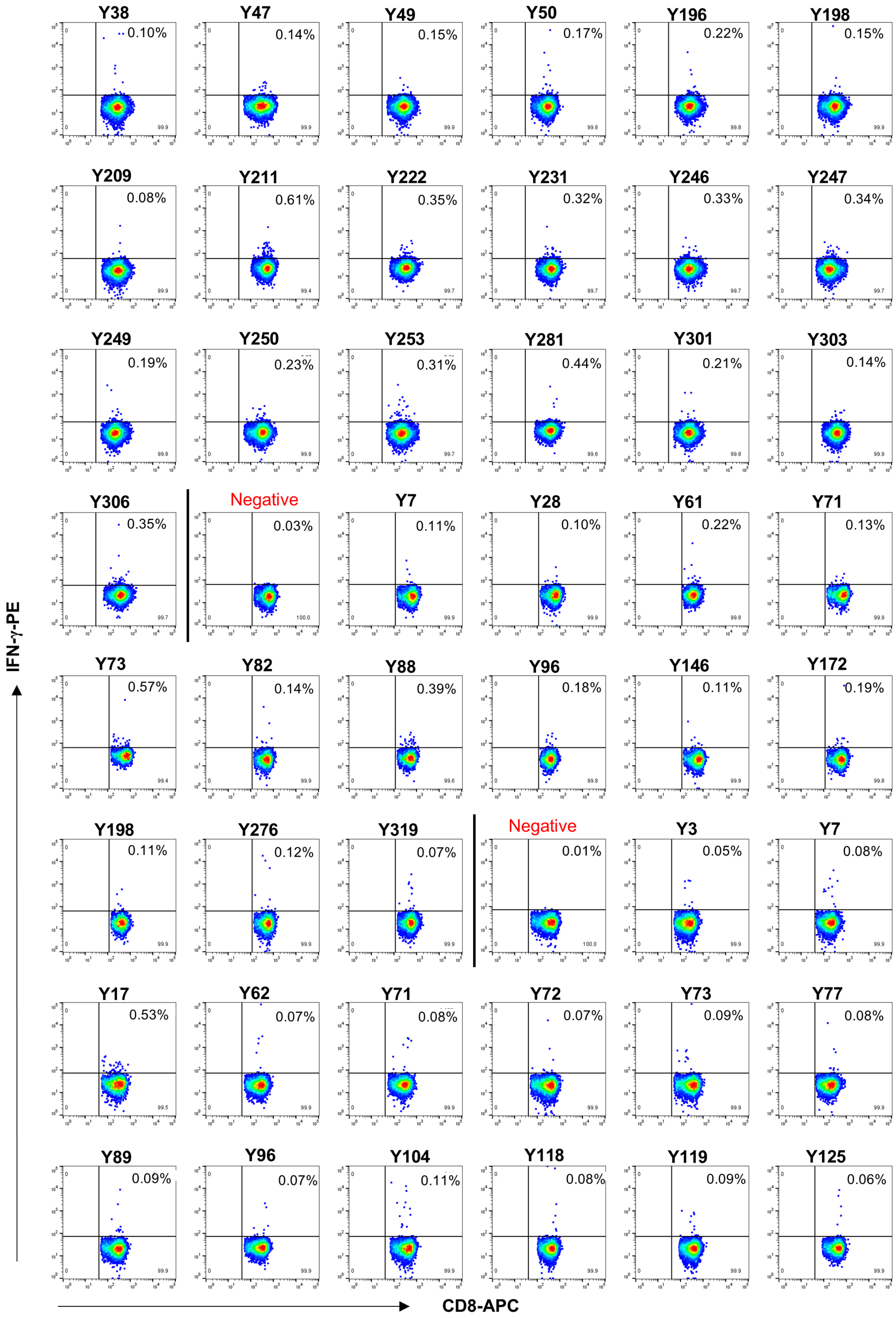
Y38 0.10%
Y47 0.14%
Y49 0.15%
Y50 0.17%
Y196 0.22%
Y198 0.15%
Y209 0.08%
Y211 0.61%
Y222 0.35%
Y231 0.32%
Y246 0.33%
Y247 0.34%
Y249 0.19%
Y250 0.23%
Y253 0.31%
Y281 0.44%
Y301 0.21%
Y303 0.14%
Y306 0.35%
Negative 0.03%
Y7 0.11%
Y28 0.10%
Y61 0.22%
Y71 0.13%
Y73 0.57%
Y82 0.14%
Y88 0.39%
Y96 0.18%
Y146 0.11%
Y172 0.19%
Y198 0.11%
Y276 0.12%
Y319 0.07%
Negative 0.01%
Y3 0.05%
Y7 0.08%
Y17 0.53%
Y62 0.07%
Y71 0.08%
Y72 0.07%
Y73 0.09%
Y77 0.08%
Y89 0.09%
Y96 0.07%
Y104 0.11%
Y118 0.08%
Y119 0.09%
Y125 0.06%
IFN-γ-PE
CD8-APC

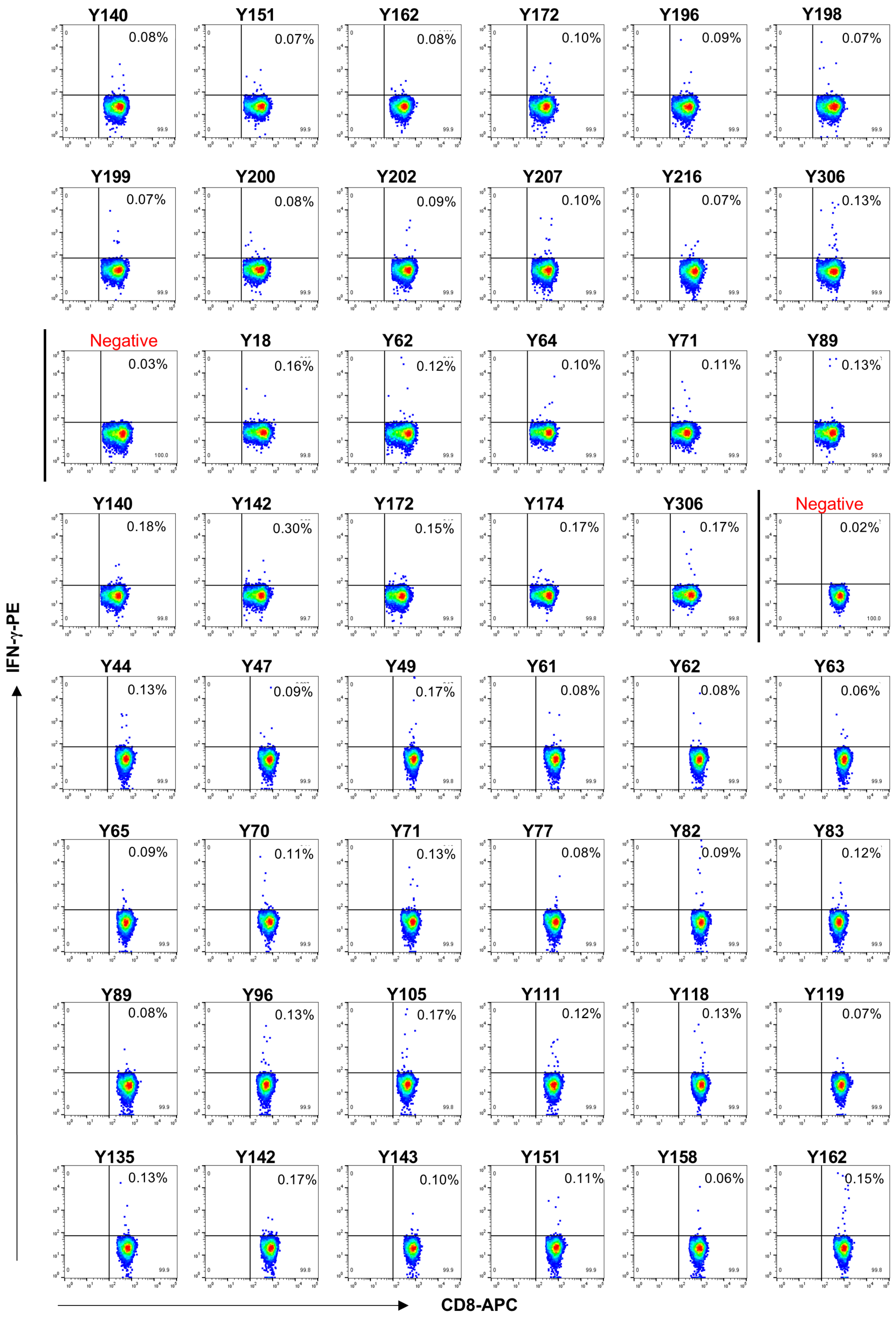
Y140
0.08%
Y151
0.07%
Y162
0.08%
Y172
0.10%
Y196
0.09%
Y198
0.07%
Y199
0.07%
Y200
0.08%
Y202
0.09%
Y207
0.10%
Y216
0.07%
Y306
0.13%
Negative
0.03%
Y18
0.16%
Y62
0.12%
Y64
0.10%
Y71
0.11%
Y89
0.13%
Y140
0.18%
Y142
0.30%
Y172
0.15%
Y174
0.17%
Y306
0.17%
Negative
0.02%
Y44
0.13%
Y47
0.09%
Y49
0.17%
Y61
0.08%
Y62
0.08%
Y63
0.06%
Y65
0.09%
Y70
0.11%
Y71
0.13%
Y77
0.08%
Y82
0.09%
Y83
0.12%
Y89
0.08%
Y96
0.13%
Y105
0.17%
Y111
0.12%
Y118
0.13%
Y119
0.07%
Y135
0.13%
Y142
0.17%
Y143
0.10%
Y151
0.11%
Y158
0.06%
Y162
0.15%
IFN-γ-PE
CD8-APC

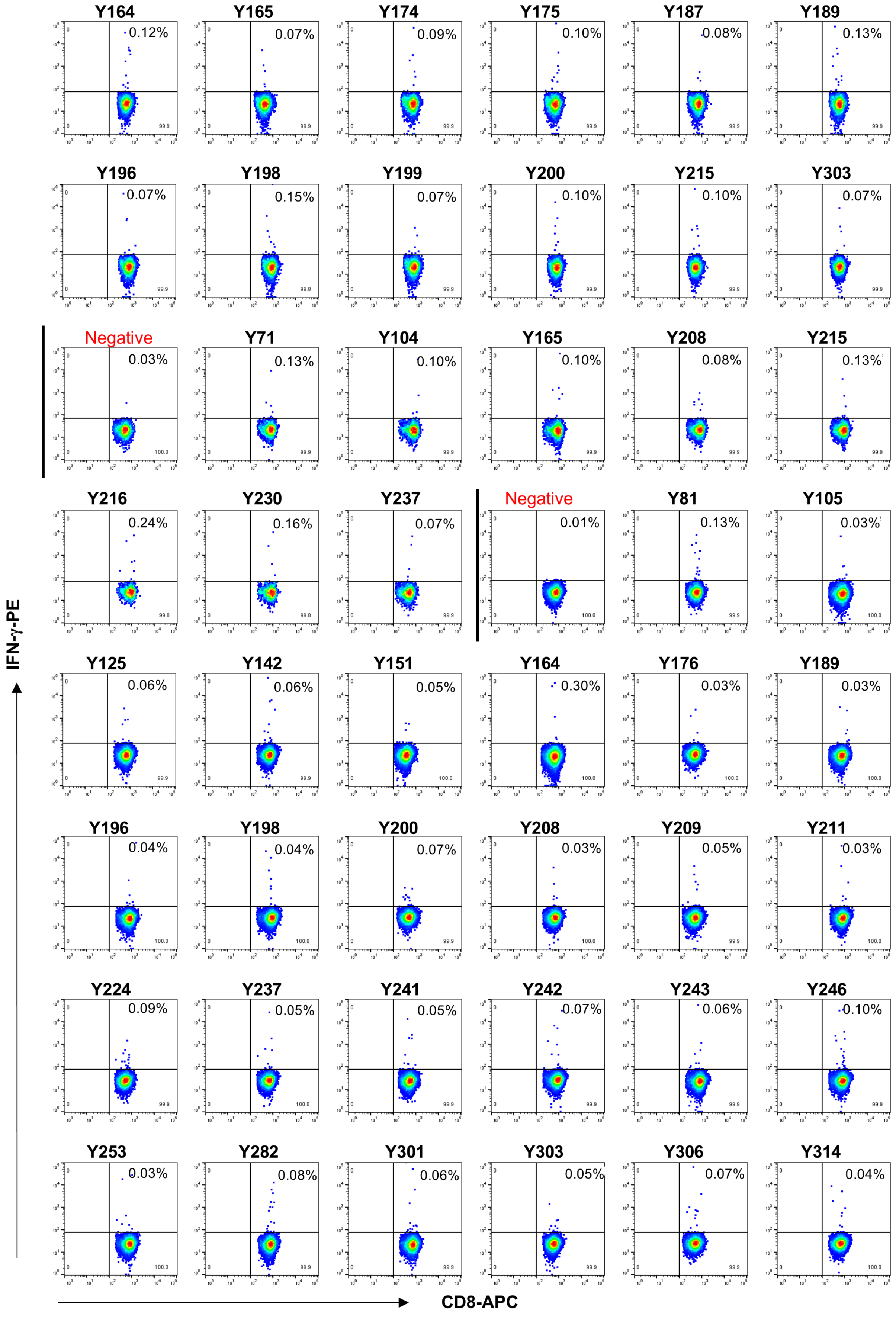

Y164
0.12%
Y165
0.07%
Y174
0.09%
Y175
0.10%
Y187
0.08%
Y189
0.13%
Y196
0.07%
Y198
0.15%
Y199
0.07%
Y200
0.10%
Y215
0.10%
Y303
0.07%
Negative
0.03%
Y71
0.13%
Y104
0.10%
Y165
0.10%
Y208
0.08%
Y215
0.13%
Y216
0.24%
Y230
0.16%
Y237
0.07%
Negative
0.01%
Y81
0.13%
Y105
0.03%
Y125
0.06%
Y142
0.06%
Y151
0.05%
Y164
0.30%
Y176
0.03%
Y189
0.03%
Y196
0.04%
Y198
0.04%
Y200
0.07%
Y208
0.03%
Y209
0.05%
Y211
0.03%
Y224
0.09%
Y237
0.05%
Y241
0.05%
Y242
0.07%
Y243
0.06%
Y246
0.10%
Y253
0.03%
Y282
0.08%
Y301
0.06%
Y303
0.05%
Y306
0.07%
Y314
0.04%
IFN-γ-PE
CD8-APC

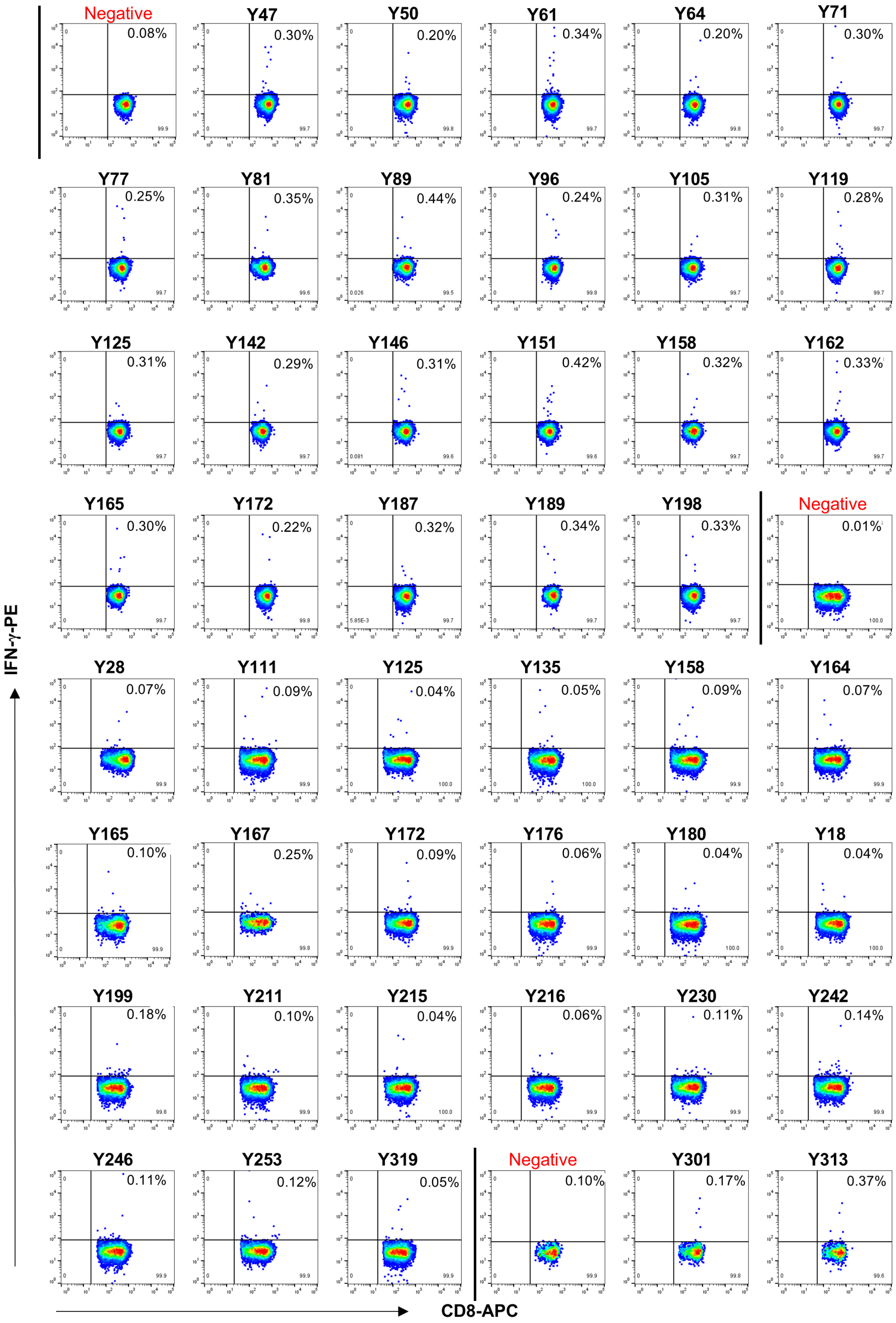
Negative
Y47
Y50
Y61
Y64
Y71
0.08%
0.30%
0.20%
0.34%
0.20%
0.30%
Y77
Y81
Y89
Y96
Y105
Y119
0.25%
0.35%
0.44%
0.24%
0.31%
0.28%
Y125
Y142
Y146
Y151
Y158
Y162
0.31%
0.29%
0.31%
0.42%
0.32%
0.33%
Y165
Y172
Y187
Y189
Y198
Negative
0.30%
0.22%
0.32%
0.34%
0.33%
0.01%
Y28
Y111
Y125
Y135
Y158
Y164
0.07%
0.09%
0.04%
0.05%
0.09%
0.07%
Y165
Y167
Y172
Y176
Y180
Y18
0.10%
0.25%
0.09%
0.06%
0.04%
0.04%
Y199
Y211
Y215
Y216
Y230
Y242
0.18%
0.10%
0.04%
0.06%
0.11%
0.14%
Y246
Y253
Y319
Negative
Y301
Y313
0.11%
0.12%
0.05%
0.10%
0.17%
0.37%
IFN-γ-PE
CD8-APC

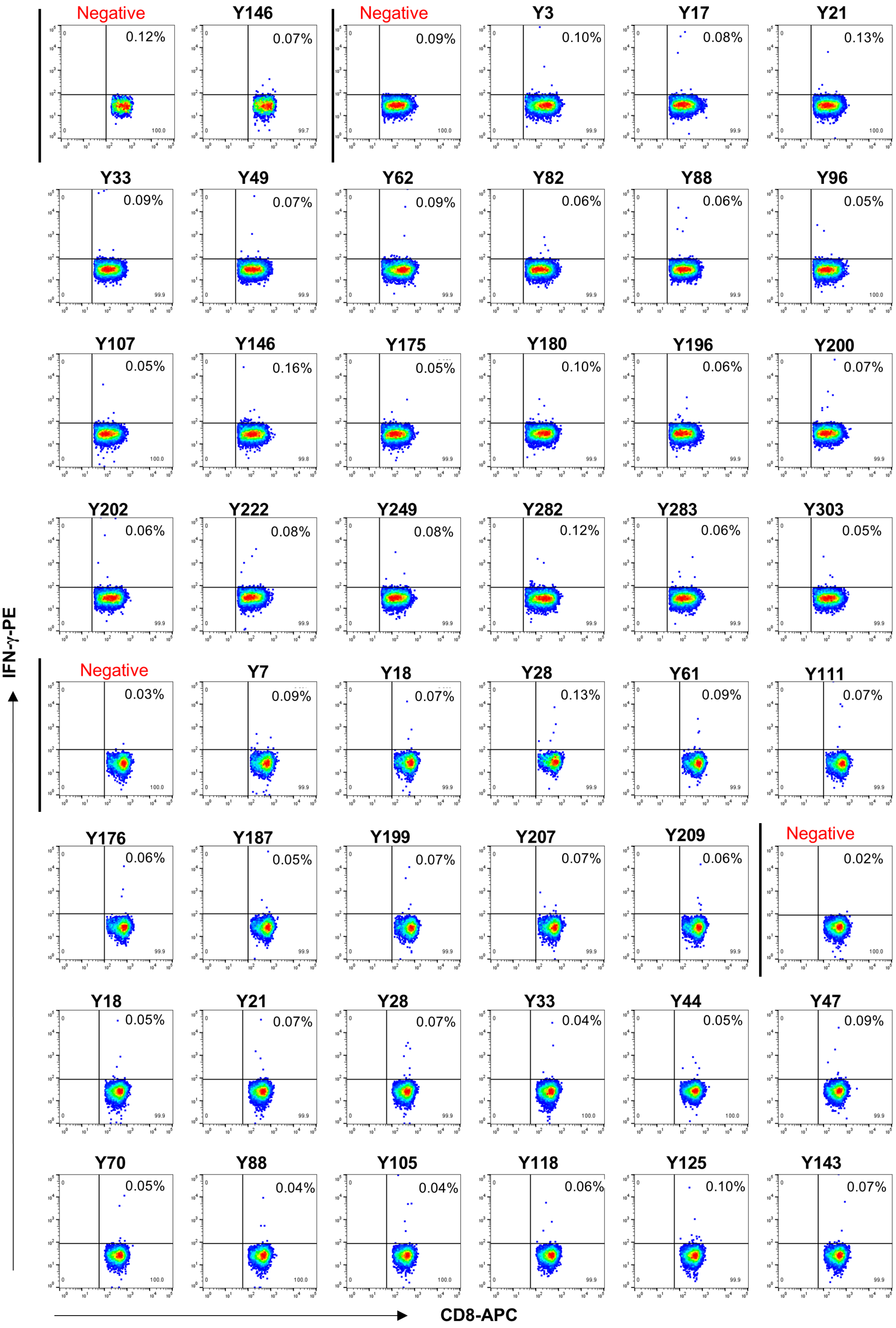

Negative
0.12%
Y146
0.07%
Negative
0.09%
Y3
0.10%
Y17
0.08%
Y21
0.13%
Y33
0.09%
Y49
0.07%
Y62
0.09%
Y82
0.06%
Y88
0.06%
Y96
0.05%
Y107
0.05%
Y146
0.16%
Y175
0.05%
Y180
0.10%
Y196
0.06%
Y200
0.07%
Y202
0.06%
Y222
0.08%
Y249
0.08%
Y282
0.12%
Y283
0.06%
Y303
0.05%
Negative
0.03%
Y7
0.09%
Y18
0.07%
Y28
0.13%
Y61
0.09%
Y111
0.07%
Y176
0.06%
Y187
0.05%
Y199
0.07%
Y207
0.07%
Y209
0.06%
Negative
0.02%
Y18
0.05%
Y21
0.07%
Y28
0.07%
Y33
0.04%
Y44
0.05%
Y47
0.09%
Y70
0.05%
Y88
0.04%
Y105
0.04%
Y118
0.06%
Y125
0.10%
Y143
0.07%
IFN-γ-PE
CD8-APC

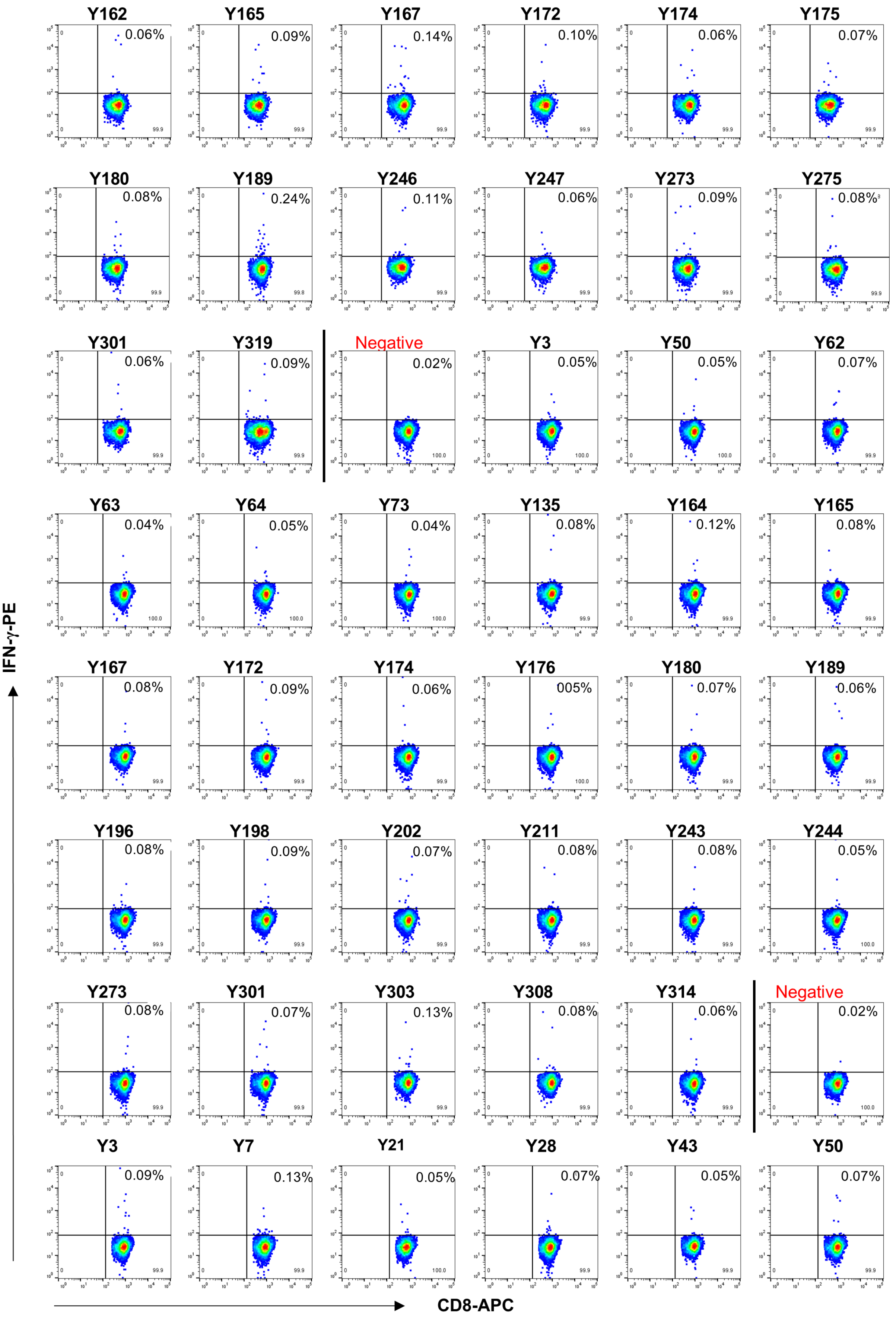
Y162
0.06%
Y165
0.09%
Y167
0.14%
Y172
0.10%
Y174
0.06%
Y175
0.07%
Y180
0.08%
Y189
0.24%
Y246
0.11%
Y247
0.06%
Y273
0.09%
Y275
0.08%
Y301
0.06%
Y319
0.09%
Negative
0.02%
Y3
0.05%
Y50
0.05%
Y62
0.07%
Y63
0.04%
Y64
0.05%
Y73
0.04%
Y135
0.08%
Y164
0.12%
Y165
0.08%
Y167
0.08%
Y172
0.09%
Y174
0.06%
Y176
005%
Y180
0.07%
Y189
0.06%
Y196
0.08%
Y198
0.09%
Y202
0.07%
Y211
0.08%
Y243
0.08%
Y244
0.05%
Y273
0.08%
Y301
0.07%
Y303
0.13%
Y308
0.08%
Y314
0.06%
Negative
0.02%
Y3
0.09%
Y7
0.13%
Y21
0.05%
Y28
0.07%
Y43
0.05%
Y50
0.07%
IFN-γ-PE
CD8-APC

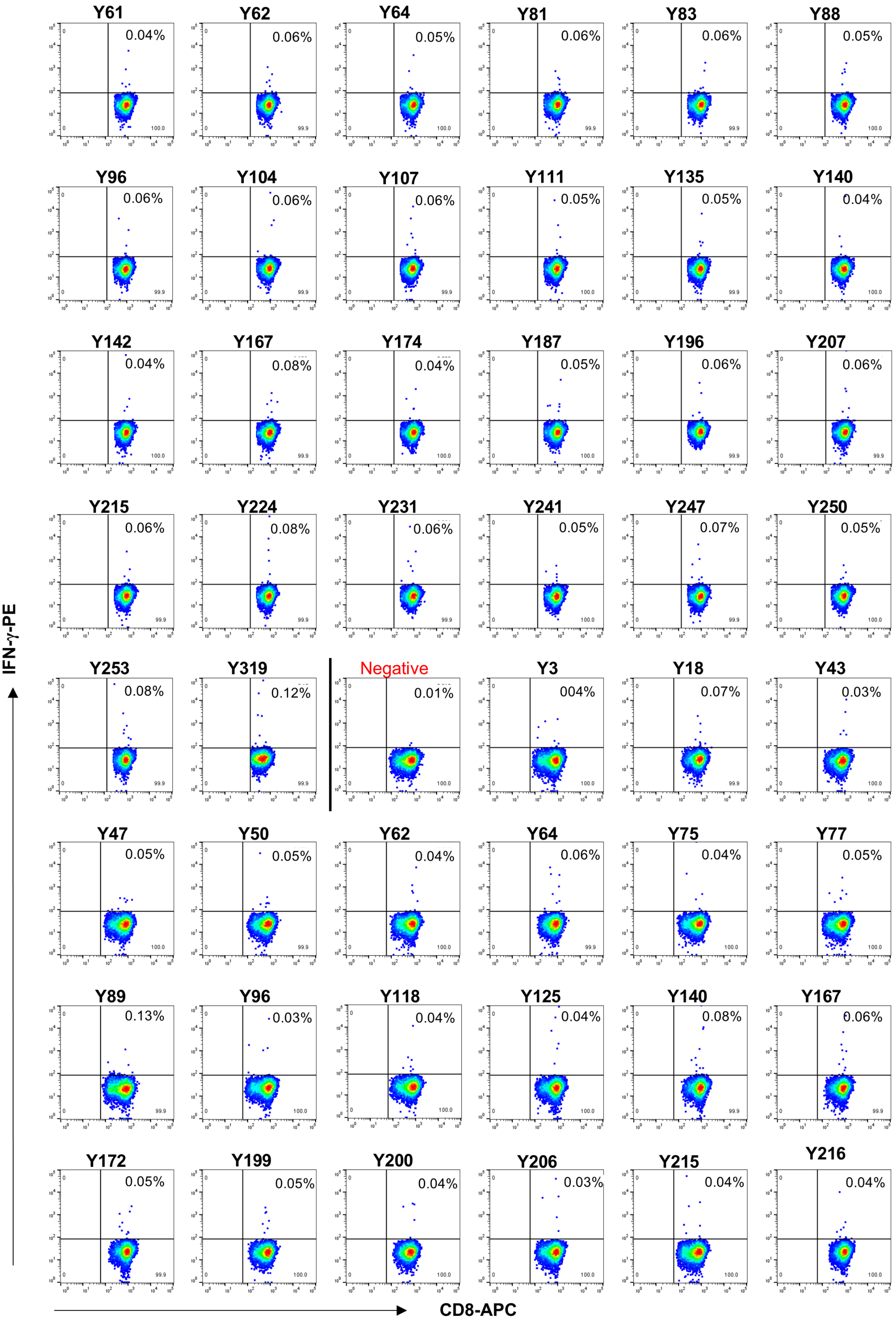
Y61 0.04%
Y62 0.06%
Y64 0.05%
Y81 0.06%
Y83 0.06%
Y88 0.05%
Y96 0.06%
Y104 0.06%
Y107 0.06%
Y111 0.05%
Y135 0.05%
Y140 0.04%
Y142 0.04%
Y167 0.08%
Y174 0.04%
Y187 0.05%
Y196 0.06%
Y207 0.06%
Y215 0.06%
Y224 0.08%
Y231 0.06%
Y241 0.05%
Y247 0.07%
Y250 0.05%
Y253 0.08%
Y319 0.12%
Negative 0.01%
Y3 004%
Y18 0.07%
Y43 0.03%
Y47 0.05%
Y50 0.05%
Y62 0.04%
Y64 0.06%
Y75 0.04%
Y77 0.05%
Y89 0.13%
Y96 0.03%
Y118 0.04%
Y125 0.04%
Y140 0.08%
Y167 0.06%
Y172 0.05%
Y199 0.05%
Y200 0.04%
Y206 0.03%
Y215 0.04%
Y216 0.04%
IFN-γ-PE
CD8-APC

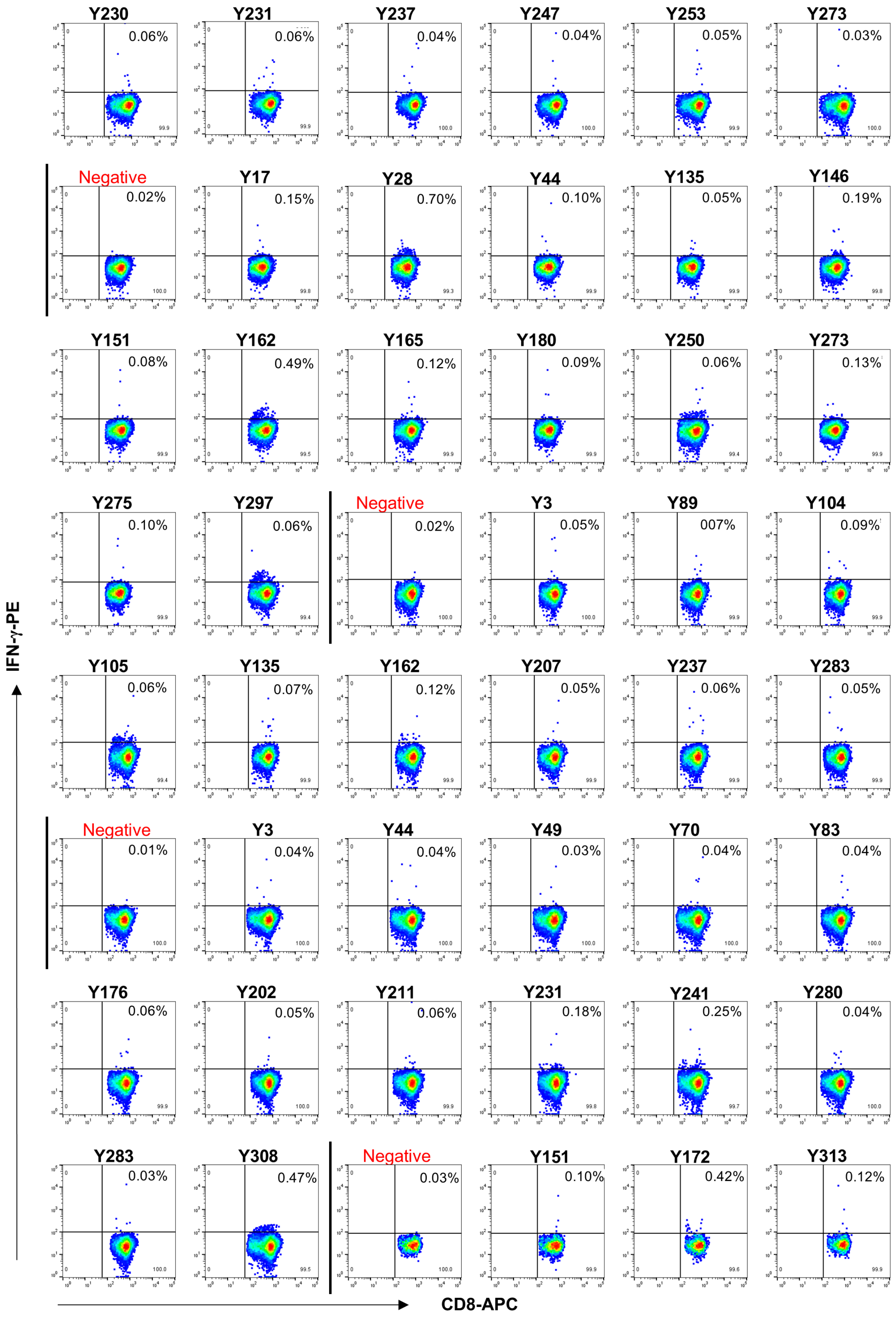
Y230
0.06%
Y231
0.06%
Y237
0.04%
Y247
0.04%
Y253
0.05%
Y273
0.03%
Negative
0.02%
Y17
0.15%
Y28
0.70%
Y44
0.10%
Y135
0.05%
Y146
0.19%
Y151
0.08%
Y162
0.49%
Y165
0.12%
Y180
0.09%
Y250
0.06%
Y273
0.13%
Y275
0.10%
Y297
0.06%
Negative
0.02%
Y3
0.05%
Y89
007%
Y104
0.09%
Y105
0.06%
Y135
0.07%
Y162
0.12%
Y207
0.05%
Y237
0.06%
Y283
0.05%
Negative
0.01%
Y3
0.04%
Y44
0.04%
Y49
0.03%
Y70
0.04%
Y83
0.04%
Y176
0.06%
Y202
0.05%
Y211
0.06%
Y231
0.18%
Y241
0.25%
Y280
0.04%
Y283
0.03%
Y308
0.47%
Negative
0.03%
Y151
0.10%
Y172
0.42%
Y313
0.12%
IFN-γ-PE
CD8-APC

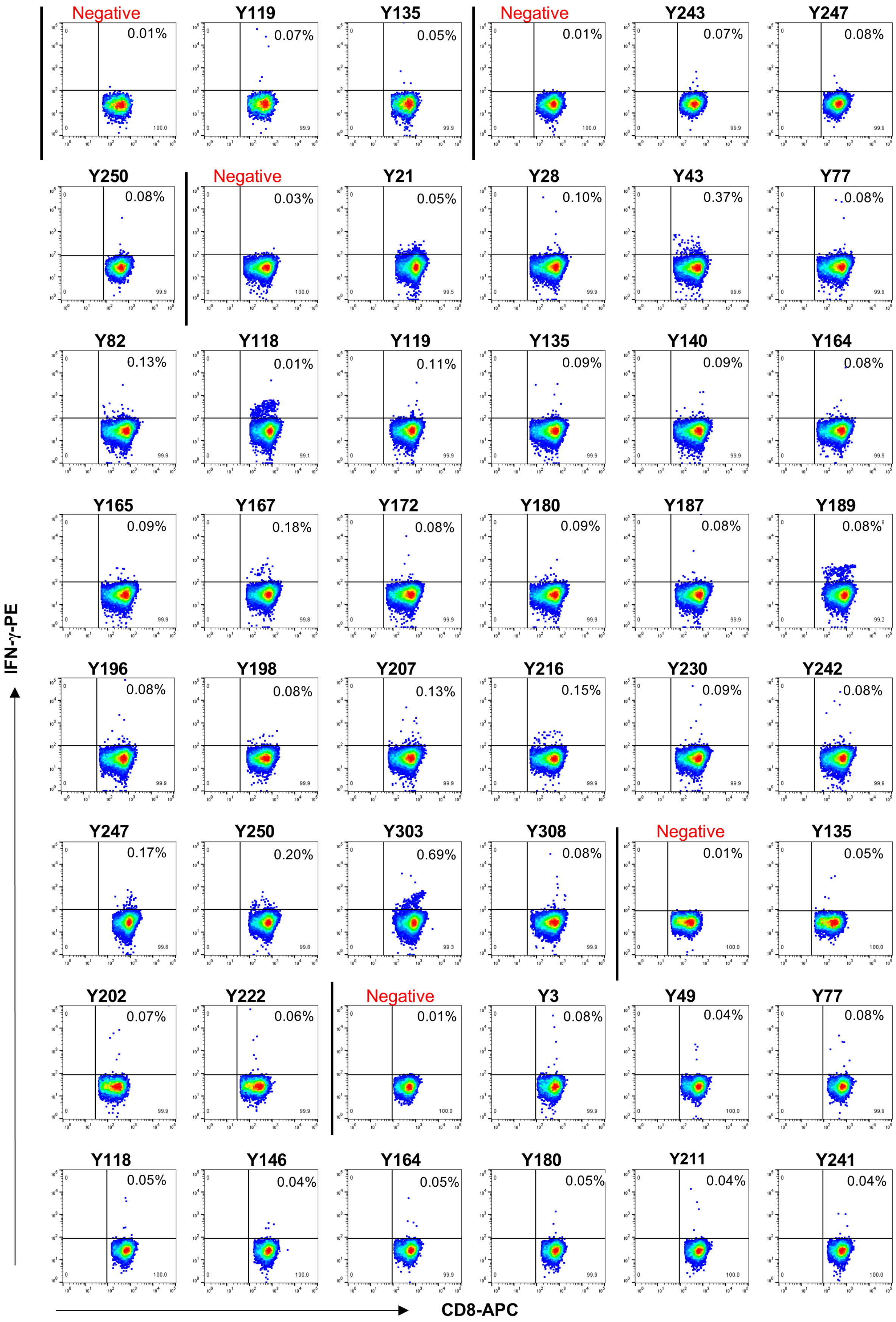
Negative 0.01%
Y119 0.07%
Y135 0.05%
Negative 0.01%
Y243 0.07%
Y247 0.08%
Y250 0.08%
Negative 0.03%
Y21 0.05%
Y28 0.10%
Y43 0.37%
Y77 0.08%
Y82 0.13%
Y118 0.01%
Y119 0.11%
Y135 0.09%
Y140 0.09%
Y164 0.08%
Y165 0.09%
Y167 0.18%
Y172 0.08%
Y180 0.09%
Y187 0.08%
Y189 0.08%
Y196 0.08%
Y198 0.08%
Y207 0.13%
Y216 0.15%
Y230 0.09%
Y242 0.08%
Y247 0.17%
Y250 0.20%
Y303 0.69%
Y308 0.08%
Negative 0.01%
Y135 0.05%
Y202 0.07%
Y222 0.06%
Negative 0.01%
Y3 0.08%
Y49 0.04%
Y77 0.08%
Y118 0.05%
Y146 0.04%
Y164 0.05%
Y180 0.05%
Y211 0.04%
Y241 0.04%
IFN-γ-PE
CD8-APC

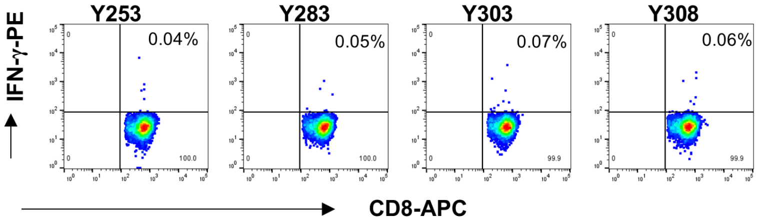

Supplementary Figure 2: The flow cytometric dot plots of intracellular IFN-γ staining for 67 HLA-B restricted epitopes which inducing CD8$^+$ T cell activation in the cocultures with 32 convalescent PBMCs samples. After *ex vivo* cocultures of PBMCs and HLA-B restricted epitope candidate peptides, the cells were harvested and followed by intracellular IFN-γ staining using FITC-conjugated anti-human CD3, APC-conjugated anti-human CD8 and PE-conjugated anti-human IFN-γ antibodies. After washing, the cells were harvested and analyzed by flow cytometry to determine the frequencies of IFN-γ$^+$ cells in CD3$^+$/CD8$^+$ populations. Negative control means PBMCs alone well; Black lines split the results of each PBMCs sample.

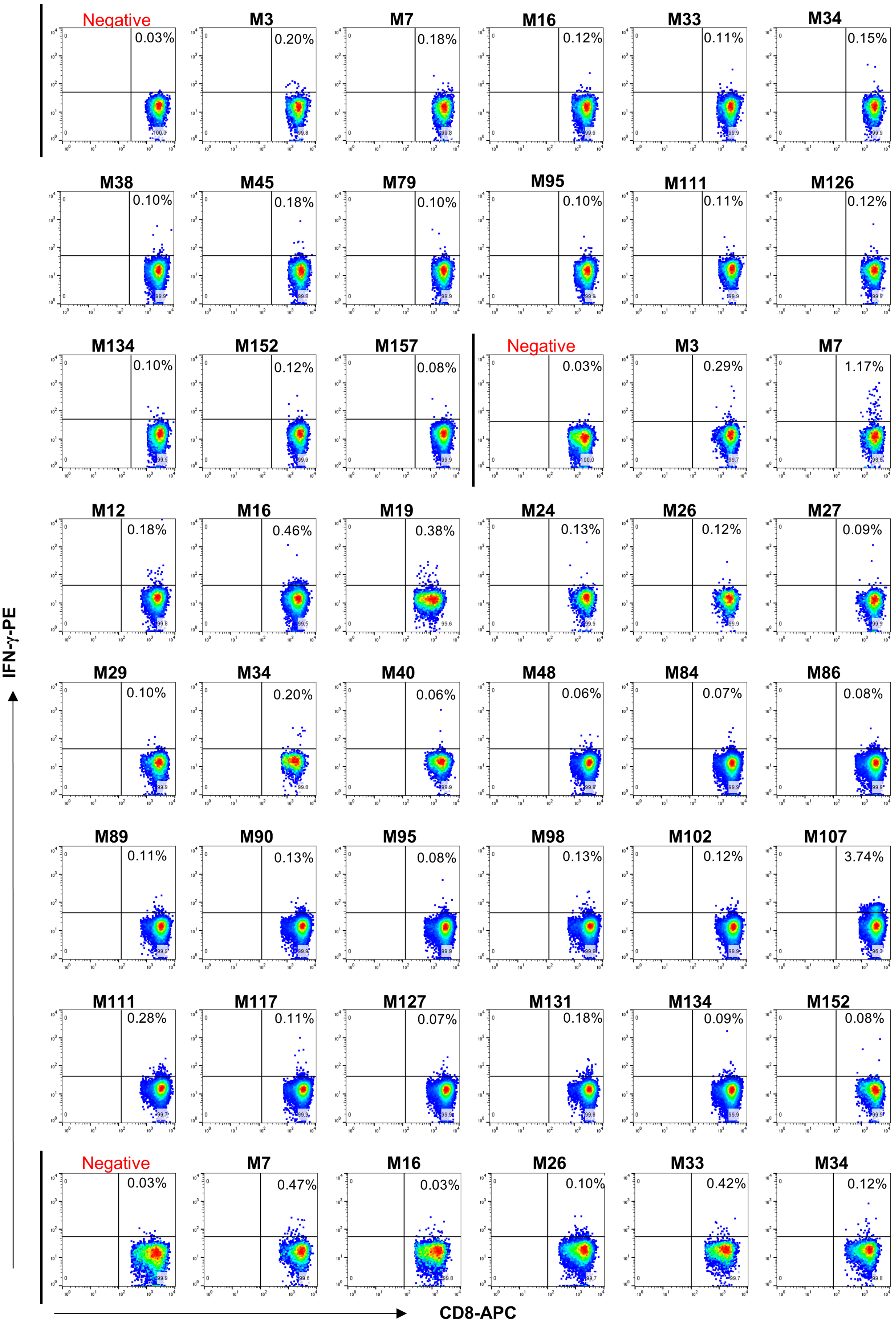
Negative
0.03%
M3
0.20%
M7
0.18%
M16
0.12%
M33
0.11%
M34
0.15%
M38
0.10%
M45
0.18%
M79
0.10%
M95
0.10%
M111
0.11%
M126
0.12%
M134
0.10%
M152
0.12%
M157
0.08%
Negative
0.03%
M3
0.29%
M7
1.17%
M12
0.18%
M16
0.46%
M19
0.38%
M24
0.13%
M26
0.12%
M27
0.09%
M29
0.10%
M34
0.20%
M40
0.06%
M48
0.06%
M84
0.07%
M86
0.08%
M89
0.11%
M90
0.13%
M95
0.08%
M98
0.13%
M102
0.12%
M107
3.74%
M111
0.28%
M117
0.11%
M127
0.07%
M131
0.18%
M134
0.09%
M152
0.08%
Negative
0.03%
M7
0.47%
M16
0.03%
M26
0.10%
M33
0.42%
M34
0.12%
IFN-γ-PE
CD8-APC

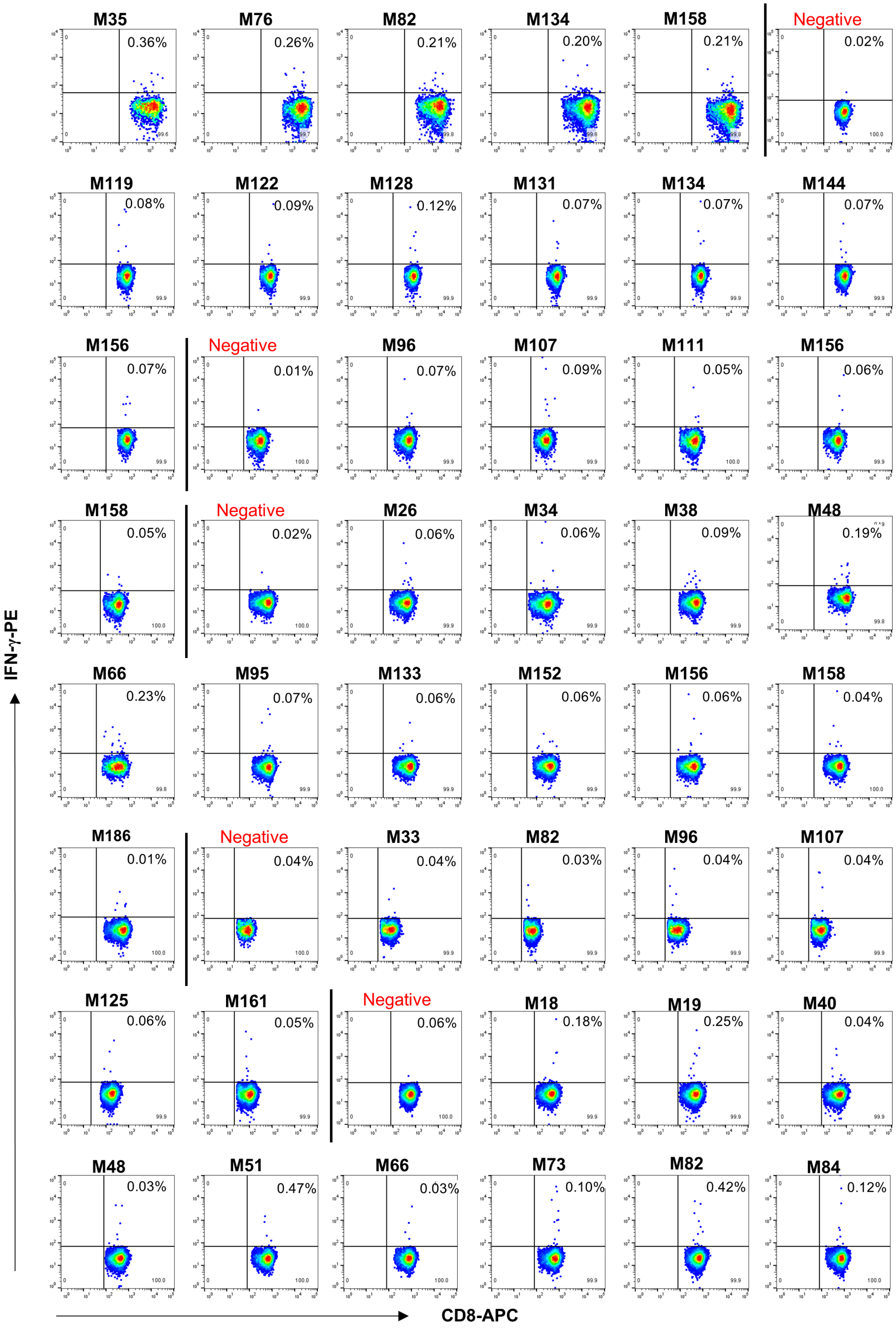

M35
0.36%
M76
0.26%
M82
0.21%
M134
0.20%
M158
0.21%
Negative
0.02%
M119
0.08%
M122
0.09%
M128
0.12%
M131
0.07%
M134
0.07%
M144
0.07%
M156
0.07%
Negative
0.01%
M96
0.07%
M107
0.09%
M111
0.05%
M156
0.06%
M158
0.05%
Negative
0.02%
M26
0.06%
M34
0.06%
M38
0.09%
M48
0.19%
M66
0.23%
M95
0.07%
M133
0.06%
M152
0.06%
M156
0.06%
M158
0.04%
M186
0.01%
Negative
0.04%
M33
0.04%
M82
0.03%
M96
0.04%
M107
0.04%
M125
0.06%
M161
0.05%
Negative
0.06%
M18
0.18%
M19
0.25%
M40
0.04%
M48
0.03%
M51
0.47%
M66
0.03%
M73
0.10%
M82
0.42%
M84
0.12%
IFN-γ-PE
CD8-APC

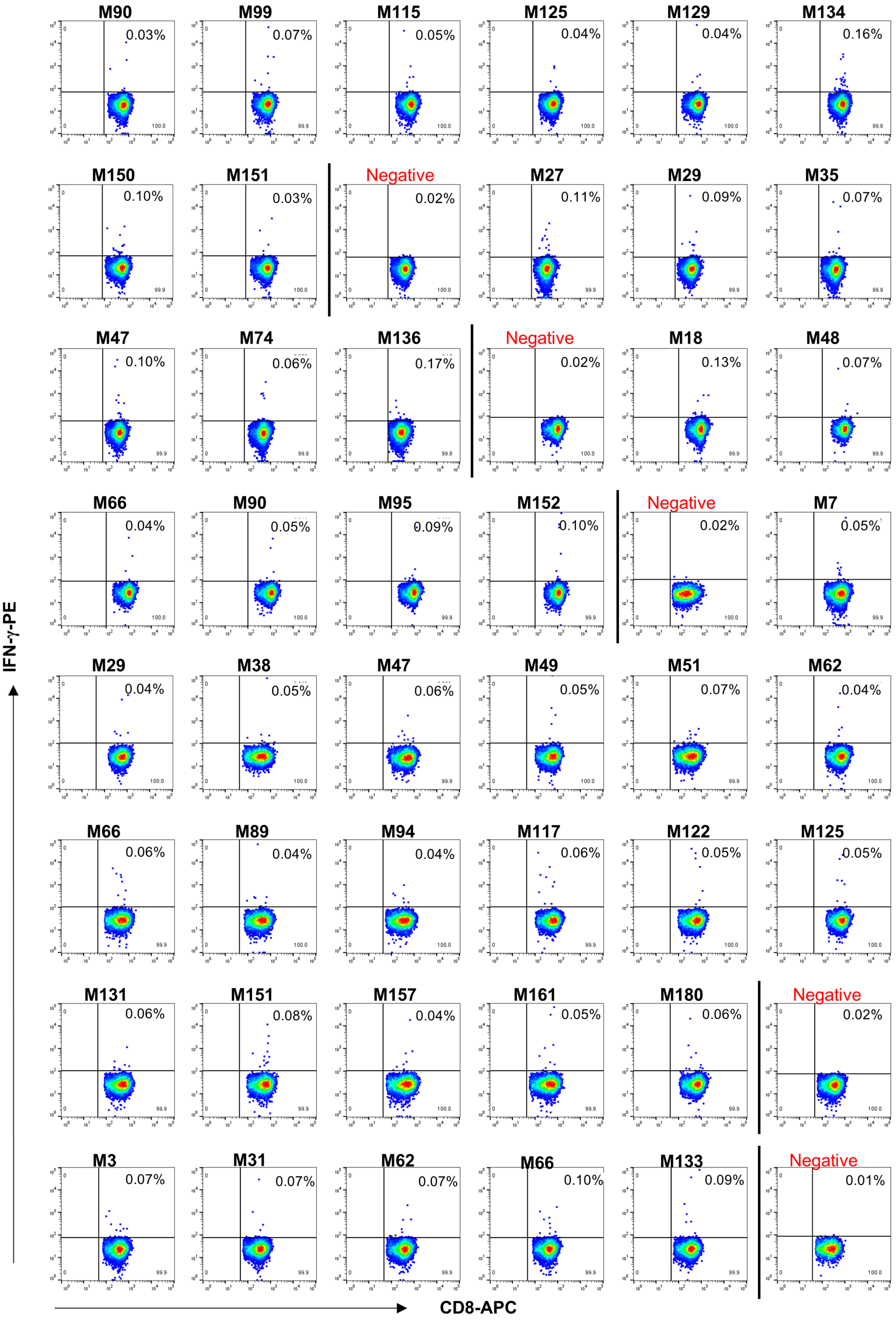
M90 0.03%
M99 0.07%
M115 0.05%
M125 0.04%
M129 0.04%
M134 0.16%
M150 0.10%
M151 0.03%
Negative 0.02%
M27 0.11%
M29 0.09%
M35 0.07%
M47 0.10%
M74 0.06%
M136 0.17%
Negative 0.02%
M18 0.13%
M48 0.07%
M66 0.04%
M90 0.05%
M95 0.09%
M152 0.10%
Negative 0.02%
M7 0.05%
M29 0.04%
M38 0.05%
M47 0.06%
M49 0.05%
M51 0.07%
M62 0.04%
M66 0.06%
M89 0.04%
M94 0.04%
M117 0.06%
M122 0.05%
M125 0.05%
M131 0.06%
M151 0.08%
M157 0.04%
M161 0.05%
M180 0.06%
Negative 0.02%
M3 0.07%
M31 0.07%
M62 0.07%
M66 0.10%
M133 0.09%
Negative 0.01%
IFN-γ-PE
CD8-APC

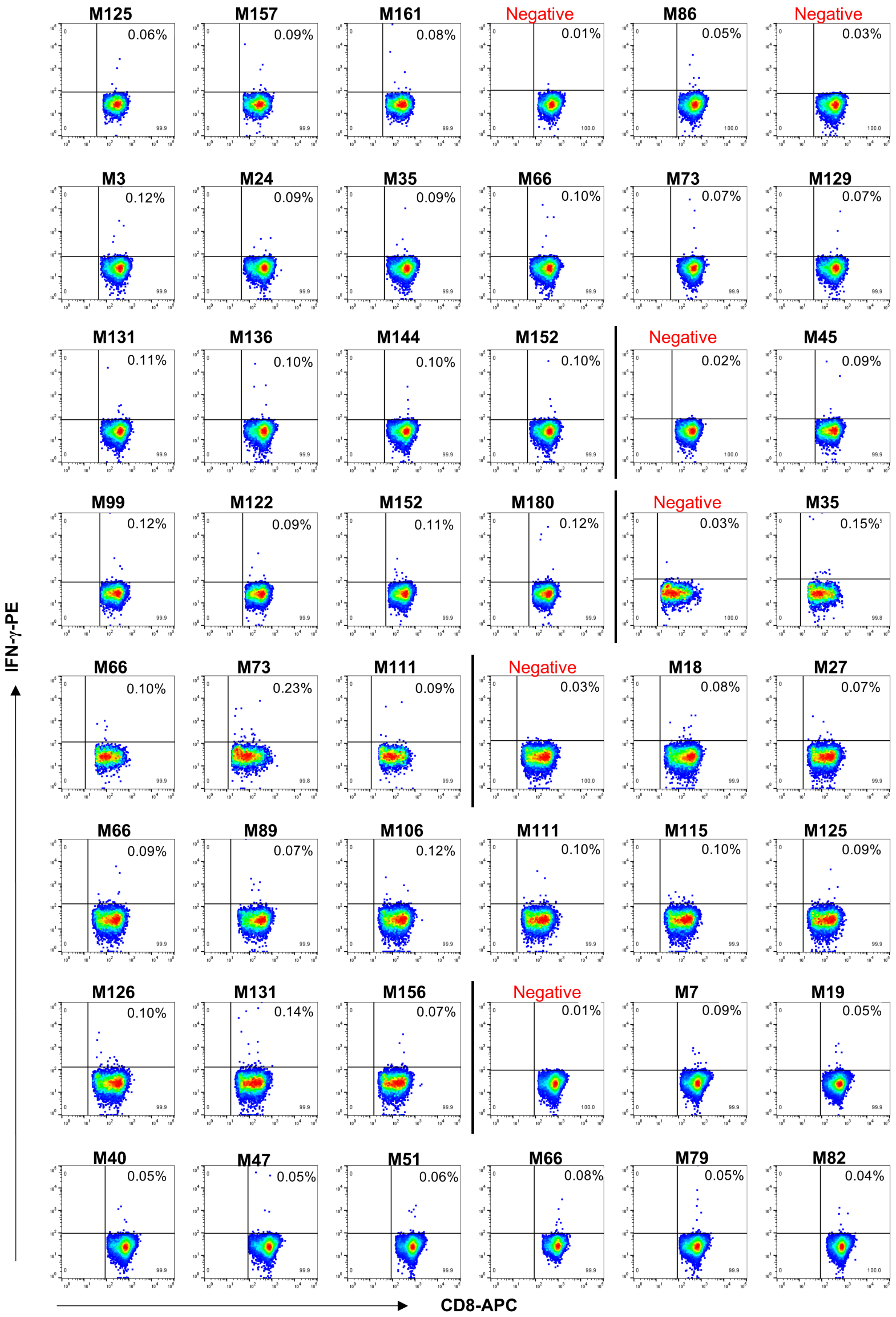
M125
0.06%
M157
0.09%
M161
0.08%
Negative
0.01%
M86
0.05%
Negative
0.03%
M3
0.12%
M24
0.09%
M35
0.09%
M66
0.10%
M73
0.07%
M129
0.07%
M131
0.11%
M136
0.10%
M144
0.10%
M152
0.10%
Negative
0.02%
M45
0.09%
M99
0.12%
M122
0.09%
M152
0.11%
M180
0.12%
Negative
0.03%
M35
0.15%
M66
0.10%
M73
0.23%
M111
0.09%
Negative
0.03%
M18
0.08%
M27
0.07%
M66
0.09%
M89
0.07%
M106
0.12%
M111
0.10%
M115
0.10%
M125
0.09%
M126
0.10%
M131
0.14%
M156
0.07%
Negative
0.01%
M7
0.09%
M19
0.05%
M40
0.05%
M47
0.05%
M51
0.06%
M66
0.08%
M79
0.05%
M82
0.04%
IFN-γ-PE
CD8-APC

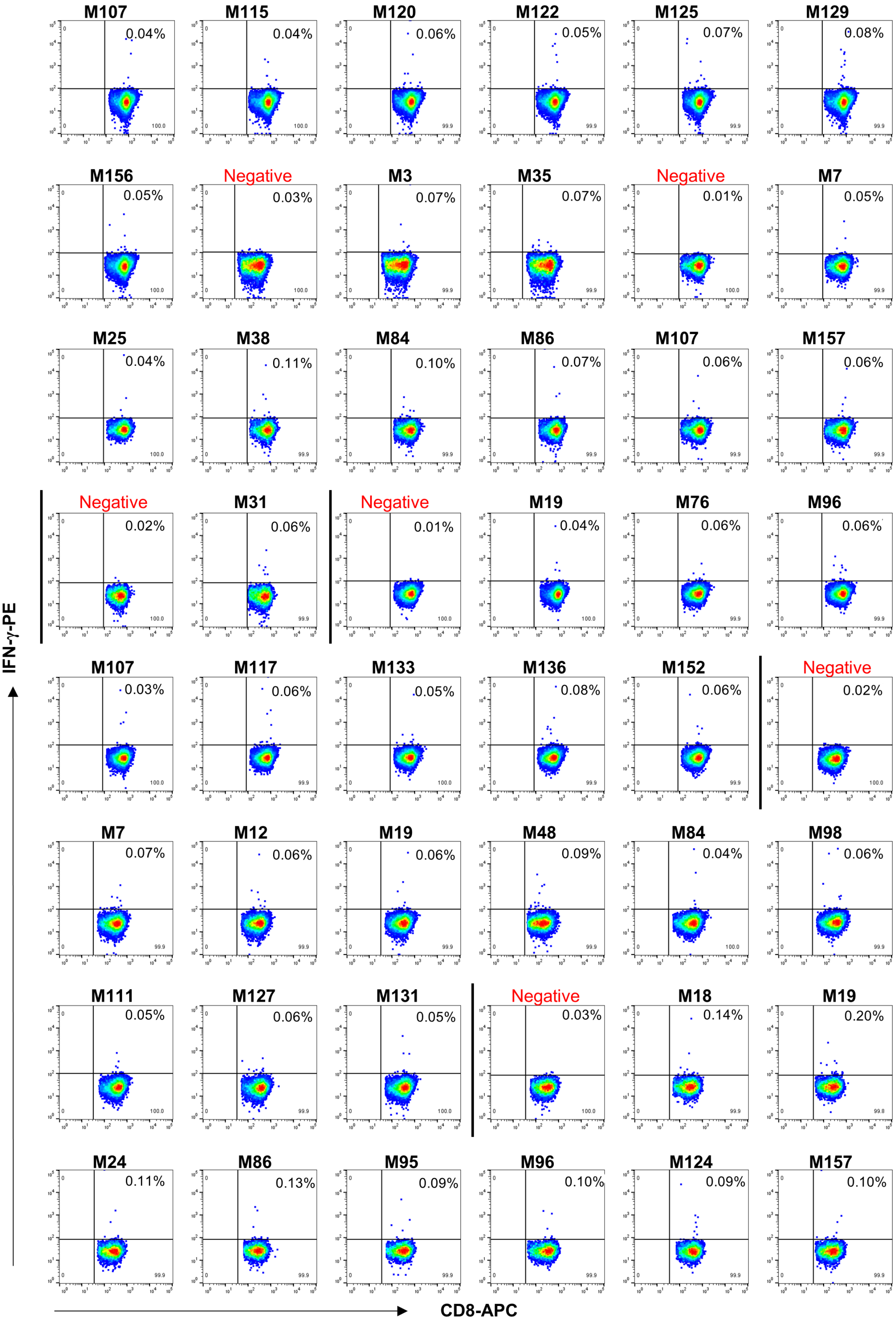
M107 0.04%
M115 0.04%
M120 0.06%
M122 0.05%
M125 0.07%
M129 0.08%
M156 0.05%
Negative 0.03%
M3 0.07%
M35 0.07%
Negative 0.01%
M7 0.05%
M25 0.04%
M38 0.11%
M84 0.10%
M86 0.07%
M107 0.06%
M157 0.06%
Negative 0.02%
M31 0.06%
Negative 0.01%
M19 0.04%
M76 0.06%
M96 0.06%
M107 0.03%
M117 0.06%
M133 0.05%
M136 0.08%
M152 0.06%
Negative 0.02%
M7 0.07%
M12 0.06%
M19 0.06%
M48 0.09%
M84 0.04%
M98 0.06%
M111 0.05%
M127 0.06%
M131 0.05%
Negative 0.03%
M18 0.14%
M19 0.20%
M24 0.11%
M86 0.13%
M95 0.09%
M96 0.10%
M124 0.09%
M157 0.10%
IFN-γ-PE
CD8-APC

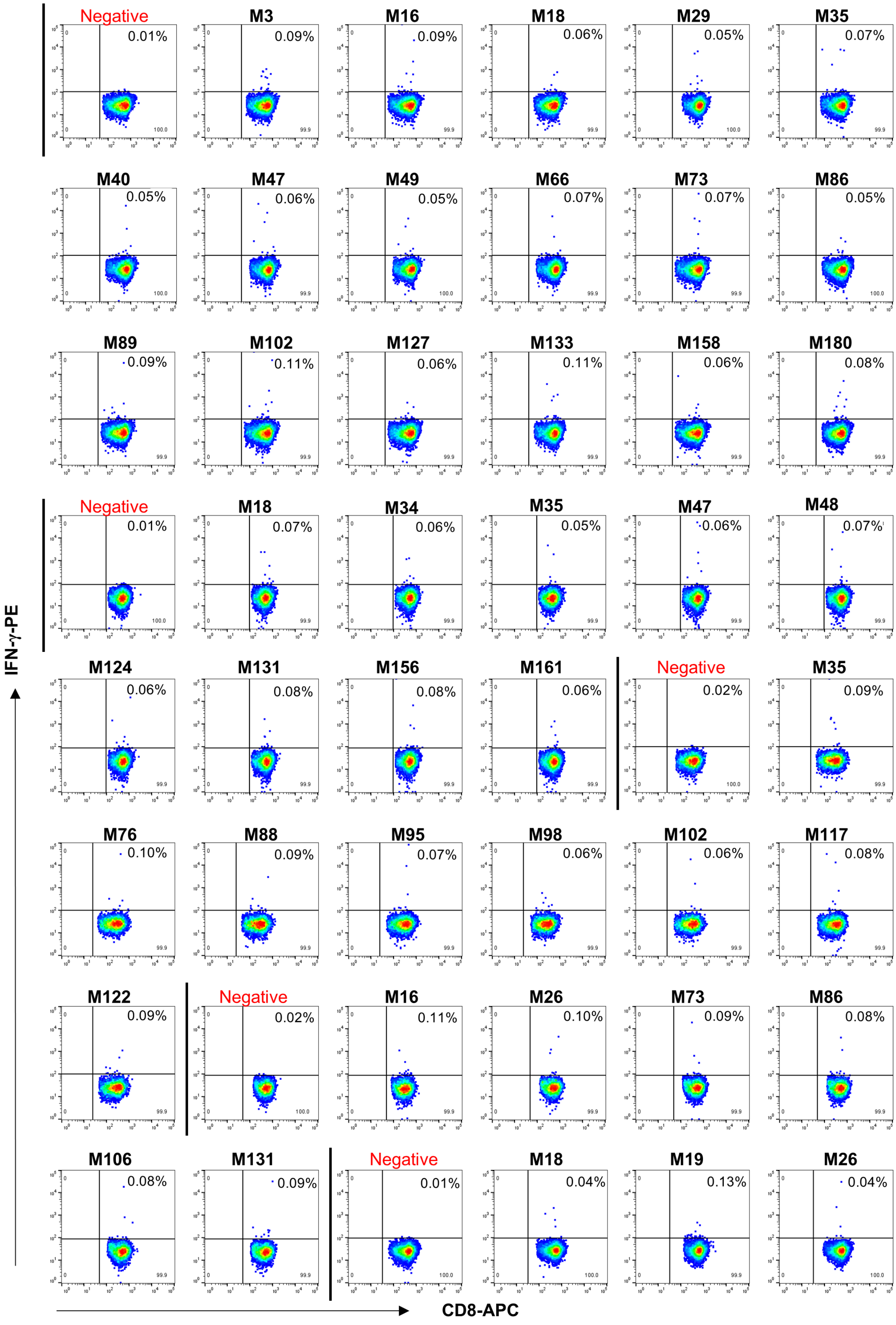

Negative
0.01%
M3
0.09%
M16
0.09%
M18
0.06%
M29
0.05%
M35
0.07%
M40
0.05%
M47
0.06%
M49
0.05%
M66
0.07%
M73
0.07%
M86
0.05%
M89
0.09%
M102
0.11%
M127
0.06%
M133
0.11%
M158
0.06%
M180
0.08%
Negative
0.01%
M18
0.07%
M34
0.06%
M35
0.05%
M47
0.06%
M48
0.07%
M124
0.06%
M131
0.08%
M156
0.08%
M161
0.06%
Negative
0.02%
M35
0.09%
M76
0.10%
M88
0.09%
M95
0.07%
M98
0.06%
M102
0.06%
M117
0.08%
M122
0.09%
Negative
0.02%
M16
0.11%
M26
0.10%
M73
0.09%
M86
0.08%
M106
0.08%
M131
0.09%
Negative
0.01%
M18
0.04%
M19
0.13%
M26
0.04%
IFN-γ-PE
CD8-APC

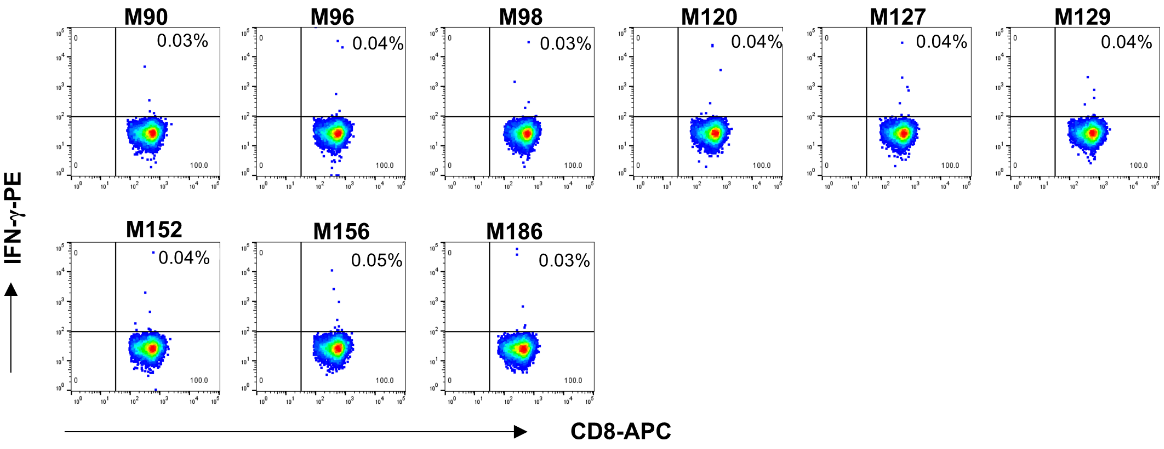

Supplementary Figure 3: The flow cytometric dot plots of intracellular IFN-γ staining for 53 HLA-C restricted epitopes which inducing CD8$^+$ T cell activation in the cocultures with 30 convalescent PBMCs samples. After *ex vivo* cocultures of PBMCs and HLA-C restricted epitope candidate peptides, the cells were harvested and followed by intracellular IFN-γ staining using FITC-conjugated anti-human CD3, APC-conjugated anti-human CD8 and PE-conjugated anti-human IFN-γ antibodies. After washing, the cells were harvested and analyzed by flow cytometry to determine the frequencies of IFN-γ$^+$ cells in CD3$^+$/CD8$^+$ populations. Negative control means PBMCs alone well; Black lines split the results of each PBMCs sample.

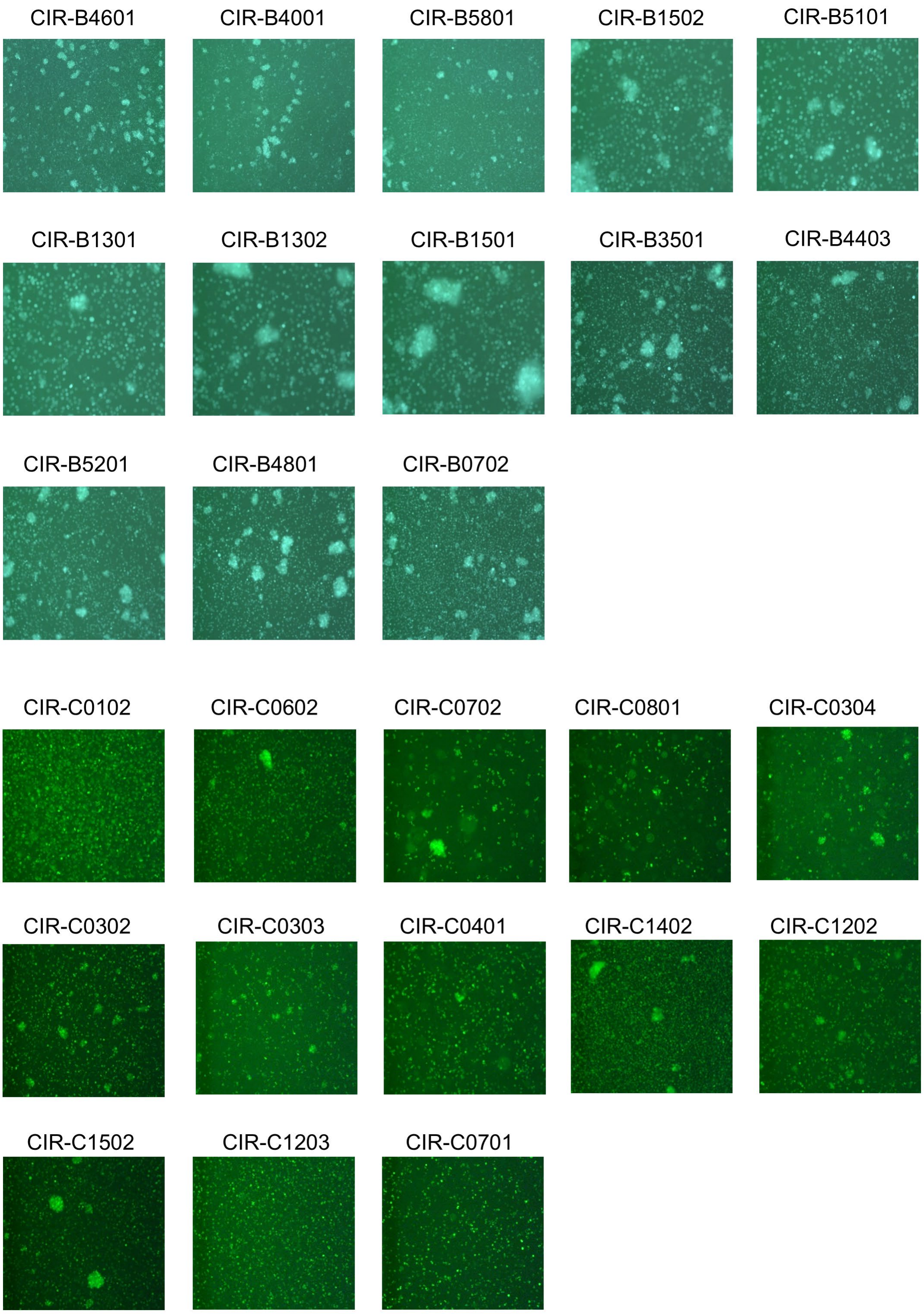

Supplementary Figure 4: Fluorescence imaging for HMy2.CIR cell lines transduced with exogenous HLA-B or HLA-C molecule. Following lentiviral infection, the HMy2.CIR cells were selected with puromycin for 72 hours. The expression of GFP in each cell line was then observed under a fluorescence microscope. Photographs were taken with a 200× magnification.

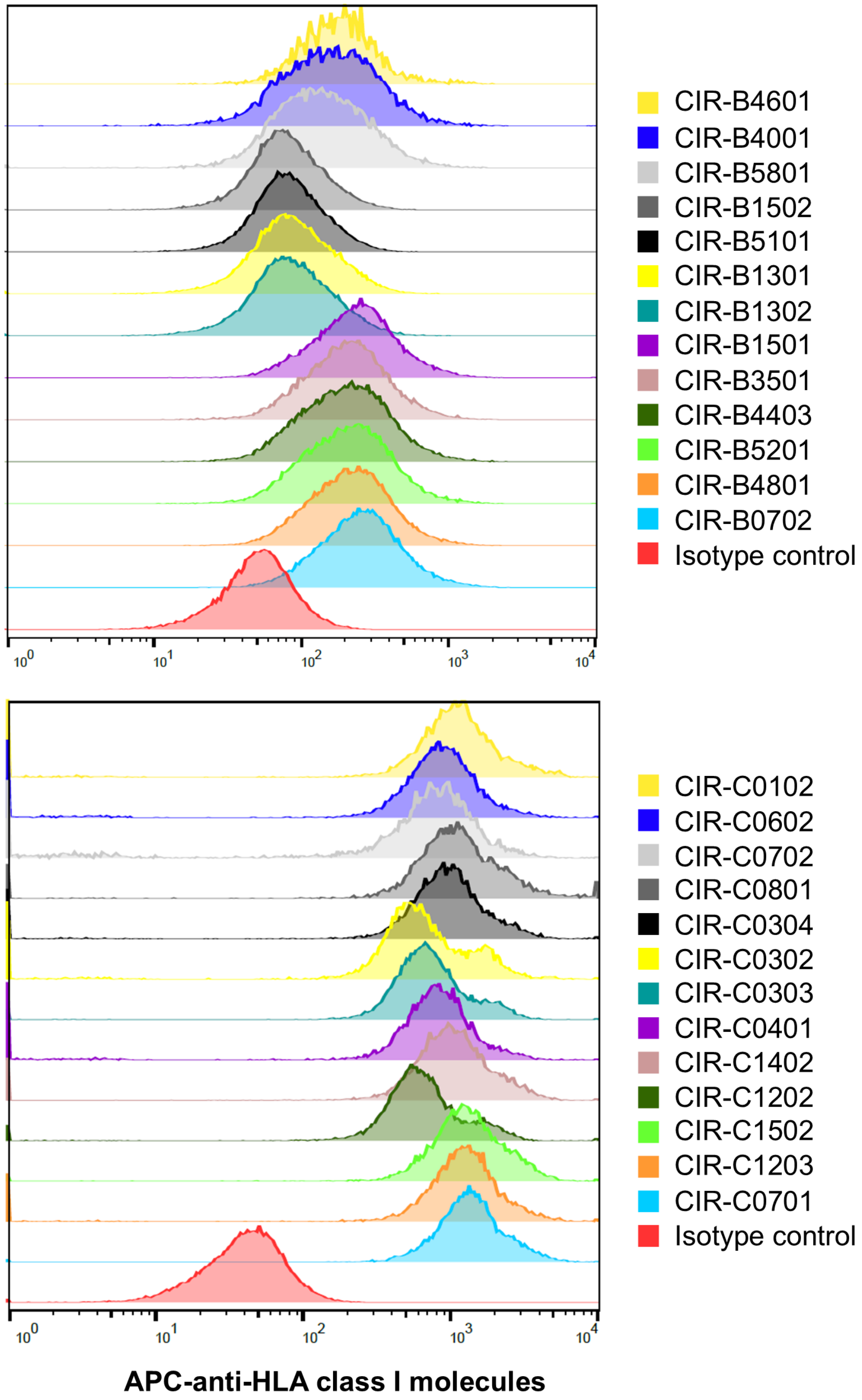

Supplementary Figure 5: The expression of HLA class I molecules onto HMy2.CIR cell lines transduced with exogenous HLA-B or HLA-C molecule. The HMy2.CIR cells transduced with exogenous HLA-B or HLA-C molecule exhibited a significantly higher fluorescence intensity compared to those transduced with the empty vector, after staining with APC-conjugated anti-HLA-A/B/C antibodies (W6/32 clone).

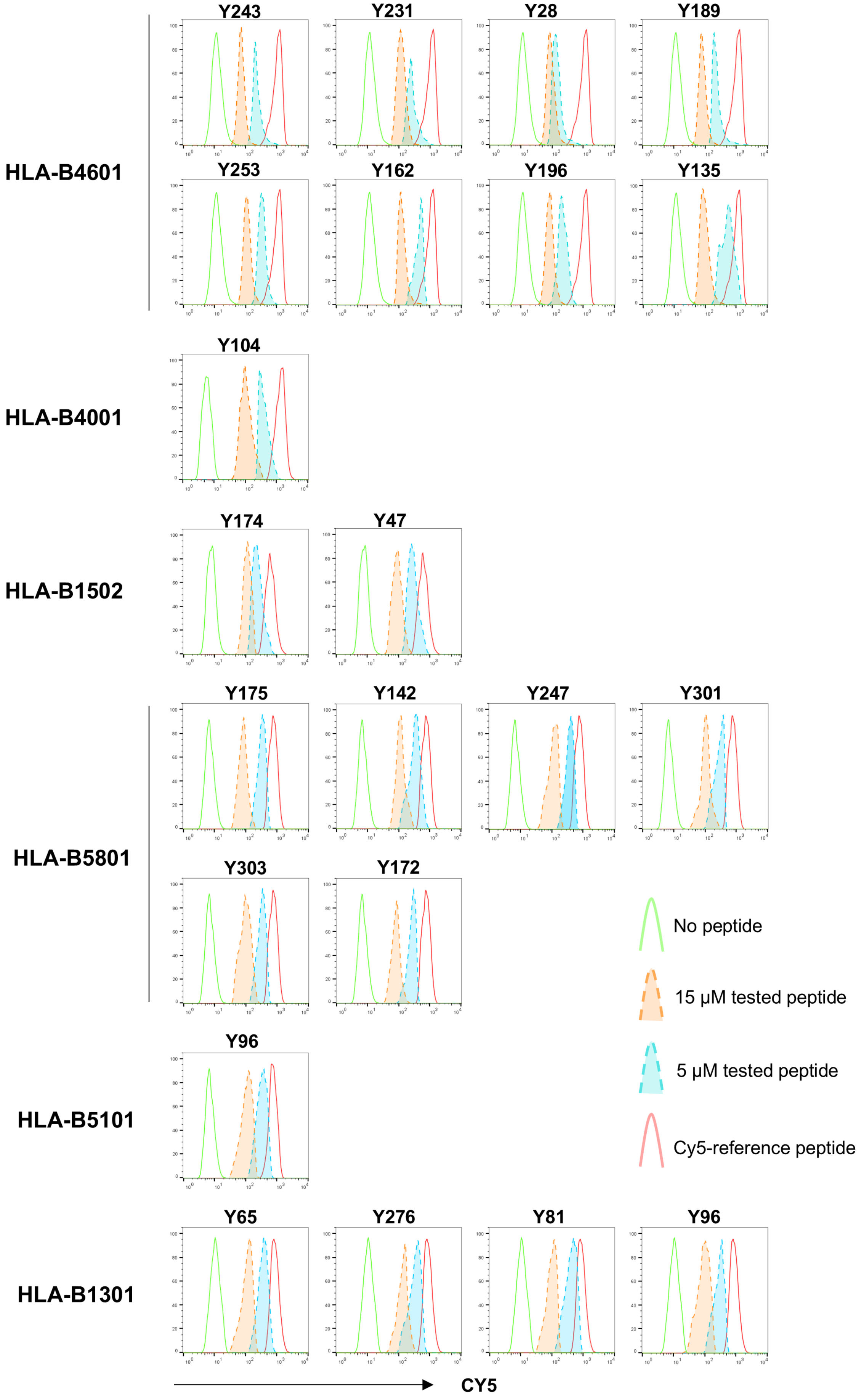
HLA-B4601
Y243
Y231
Y28
Y189
Y253
Y162
Y196
Y135
HLA-B4001
Y104
HLA-B1502
Y174
Y47
HLA-B5801
Y175
Y142
Y247
Y301
Y303
Y172
HLA-B5101
Y96
HLA-B1301
Y65
Y276
Y81
Y96
No peptide
15 μM tested peptide
5 μM tested peptide
Cy5-reference peptide
CY5

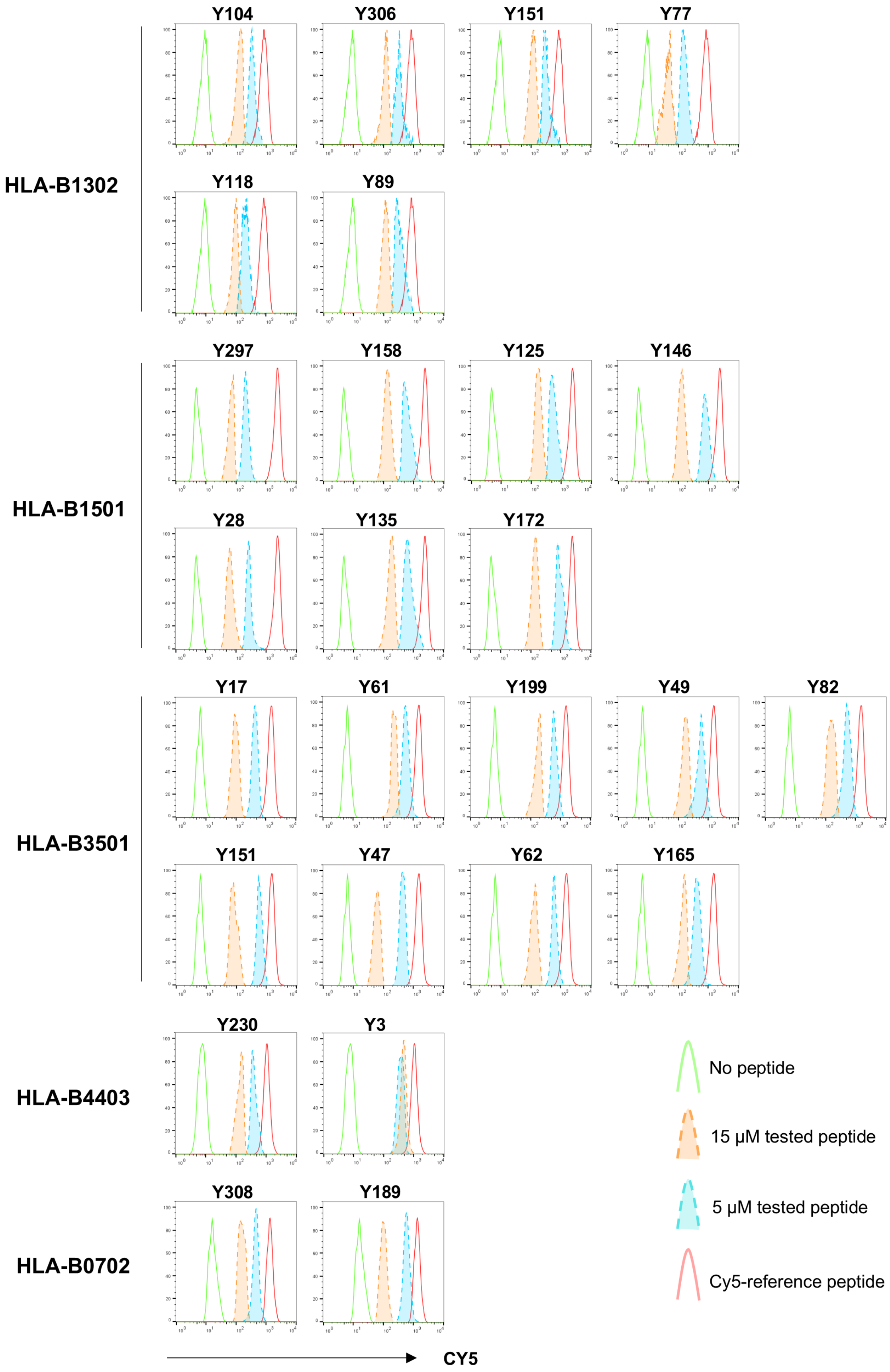

HLA-B1302
Y104
Y306
Y151
Y77
Y118
Y89
HLA-B1501
Y297
Y158
Y125
Y146
Y28
Y135
Y172
HLA-B3501
Y17
Y61
Y199
Y49
Y82
Y151
Y47
Y62
Y165
HLA-B4403
Y230
Y3
HLA-B0702
Y308
Y189
CY5
No peptide
15 μM tested peptide
5 μM tested peptide
Cy5-reference peptide

Supplementary Figure 6: Competitive peptide binding results of each high-affinity epitope restricted by each HLA-B allotype. The HMy2.CIR cells constantly expressing indicated HLA-B molecule were incubated with Cy5-labeled reference peptide and the no-labeled epitope peptide. After a defined incubation period, the unbound peptides were removed, and the relative binding affinity of the tested epitope peptide to the HLA-B molecule was quantified by the declined MFI of reference peptide binding to the CIR cell surface at different concentration of tested epitope peptide (5μm and 15μm). In parallel, the max control well (CIR cells and Cy5-reference peptide; Red solid-line peak in flow cytometry histograms) and background well (CIR cells alone; Green solid-line peak in flow cytometry histograms) were performed.

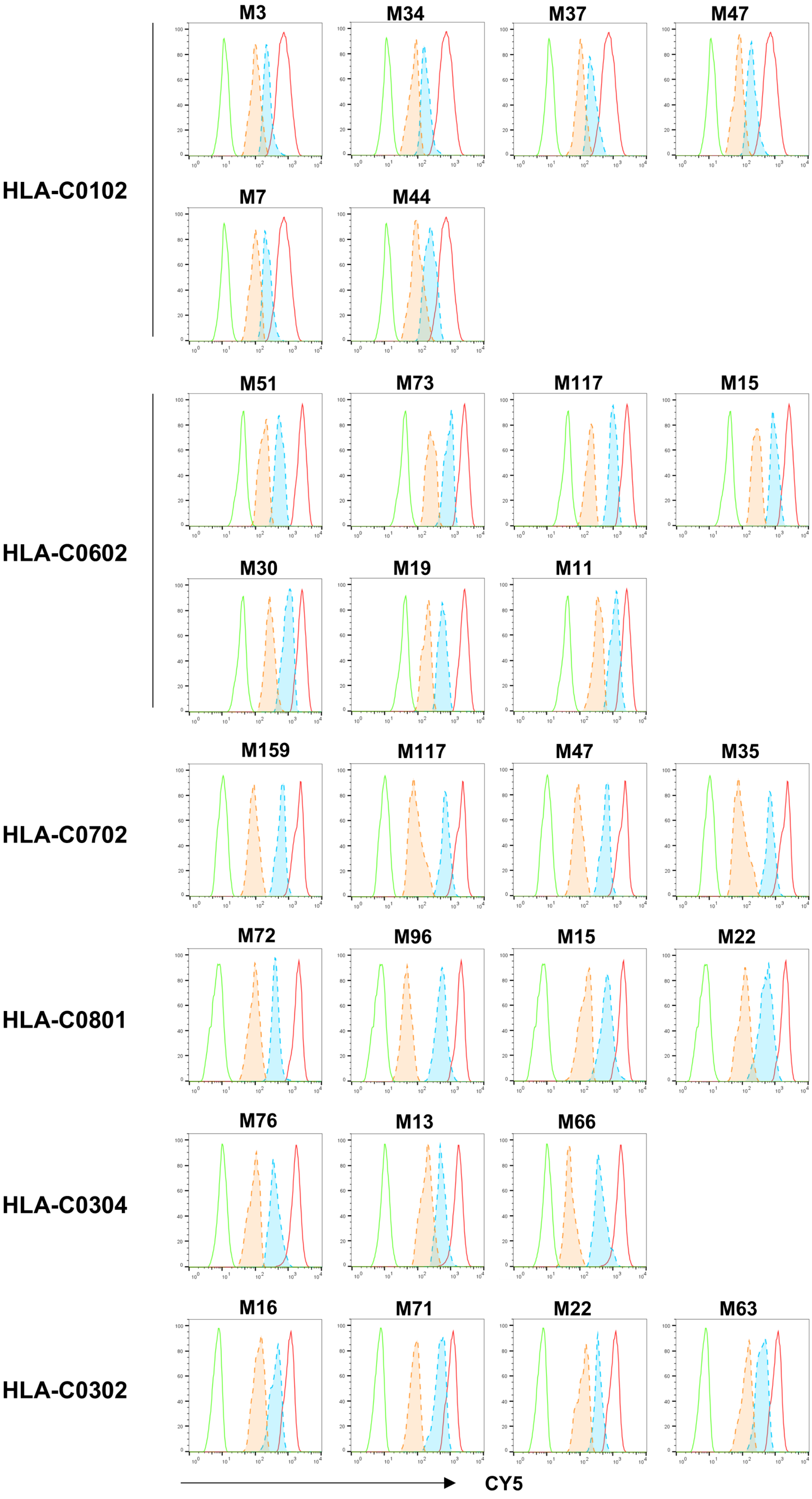
HLA-C0102
M3
M34
M37
M47
M7
M44
HLA-C0602
M51
M73
M117
M15
M30
M19
M11
HLA-C0702
M159
M117
M47
M35
HLA-C0801
M72
M96
M15
M22
HLA-C0304
M76
M13
M66
HLA-C0302
M16
M71
M22
M63
CY5

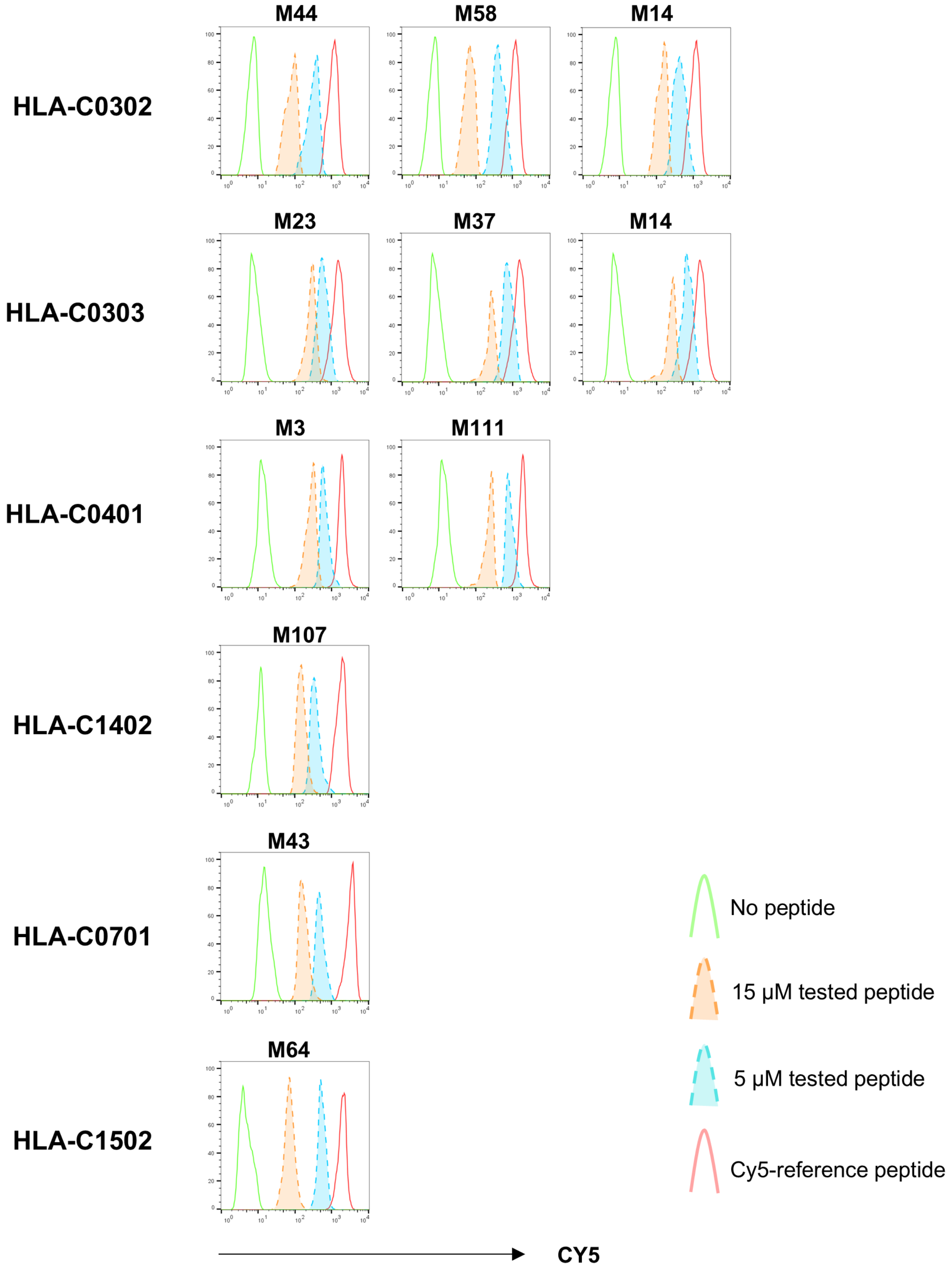

Supplementary Figure 7: Competitive peptide binding results of each high-affinity epitope restricted by each HLA-C allotype. The HMy2.CIR cells constantly expressing indicated HLA-C molecule were incubated with Cy5-labeled reference peptide and the no-labeled epitope peptide. After a defined incubation period, the unbound peptides were

removed, and the relative binding affinity of the tested epitope peptide to the HLA-C molecule was quantified by the declined MFI of reference peptide binding to the CIR cell surface at different concentration of tested epitope peptide (5μm and 15μm). In parallel, the max control well (CIR cells and Cy5-reference peptide; Red solid-line peak in flow cytometry histograms) and background well (CIR cells alone; Green solid-line peak in flow cytometry histograms) were performed.

Supplementary tables

Table S1: A total of 101 HLA-B restricted $CD8^+$ T-cell epitope candidates were synthesized for further validation.

ID	Sequence	HLA-B allotypes binding to each epitope candidate as *in silico* predicted	Protein	Start	End
**Y33**	FPQSAPHGVV	B0702, B5101	S	1,052	1,061
**Y38**	MIAQYTSAL	B0702, B1502, B4601	S	869	877
**Y81**	LQIPFAMQM	B1301, B1501, B4801	S	894	902
**Y82**	KQIYKTPPI	B1301, B1302, B4801, B5201	S	786	794
**Y83**	FEYVSQPFL	B1301, B4001, B4801	S	168	176
**Y86**	RSFIEDLLF	B1301, B5801	S	815	823
**Y88**	LQYGSFCTQL	B1301, B1501, B4801, B5201	S	754	763
**Y89**	FQTRAGCLI	B1301, B1302, B5201	S	643	651
**Y118**	LQTYVTQQLI	B1302, B5201	S	1,004	1,013
**Y119**	KEIDRLNEV	B1302, B4001	S	1,181	1,189
**Y146**	QSAPHGVVF	B1501, B4601	S	1,054	1,062
**Y151**	VASQSIIAY	B1501, B1502, B3501, B4601	S	687	695
**Y172**	FVFKNIDGY	B1301, B1302, B1501, B1502, B3501, B4403, B4601, B4801, B5101, B5201, B5801	S	192	200
**Y174**	WTAGAAAYY	B1502	S	258	266
**Y175**	QLTPTWRVY	B1502	S	628	636
**Y187**	LPFNDGVYF	B3501, B5101	S	84	92
**Y189**	FAMQMAYRF	B3501, B4601, B5801	S	898	906
**Y206**	AEVQIDRLI	B4001, B4403	S	989	997
**Y207**	NESLIDLQEL	B4001	S	1194	1203
**Y208**	VEAEVQIDRL	B4001	S	987	996
**Y209**	FERDISTEI	B4001, B5201	S	464	472
**Y230**	YEQYIKWPW	B4403	S	1206	1214
**Y231**	TECSNLLLQY	B4403	S	747	756
**Y247**	YSSANNCTF	B4601, B5801	S	160	168
**Y249**	STQDLFLPF	B4601	S	50	58
**Y250**	FVSNGTHWF	B4601	S	1095	1103

**Y280**	LPLVSSQCV	B5101	S	8	16
**Y281**	FPQSAPHGV	B5101	S	1052	1060
**Y282**	LPFQQFGRDI	B5101	S	560	569
**Y283**	IANQFNSAI	B5101, B5201	S	923	931
**Y313**	HADQLTPTW	B5801	S	625	633
**Y314**	LAGTITSGW	B5801	S	878	886
**Y21**	MPILTLTRAL	B0702, B3501, B5101	R	242	251
**Y28**	YLRKHFSMM	B0702, B1501, B4601	R	748	756
**Y70**	TQMNLKYAI	B1301, B1302, B4801, B5201	R	540	548
**Y71**	QEYADVFHL	B1301, B1302, B4001, B4403	R	875	883
**Y72**	RQLLFVVEV	B1301, B1302, B4801, B5201	R	467	475
**Y73**	HEFCSQHTM	B1301, B1302, B4001, B4403	R	810	818
**Y75**	YQHEETIYNL	B1301, B1302, B4801	R	80	89
**Y77**	RAMPNMLRI	B1301, B1302, B5101, B5201, B5801	R	624	632
**Y111**	AMYTPHTVL	B1302, B4801, B5201	R	923	931
**Y140**	MVMCGGSLY	B1501, B1502, B3501, B4601	R	666	674
**Y142**	LSFKELLVY	B1501, B4601, B5801	R	366	374
**Y143**	LMIERFVSL	B1501, B4601, B4801, B5201	R	854	862
**Y164**	HMLDMYSVM	B1502	R	898	906
**Y165**	LVYAADPAM	B1502, B3501, B4601	R	372	380
**Y166**	FVSLAIDAY	B1502, B3501	R	859	867
**Y167**	FVVSTGYHF	B1502, B3501, B4601	R	340	348
**Y198**	AESHVDTDL	B4001, B4403	R	253	261
**Y199**	HEETIYNLL	B4001	R	82	90
**Y200**	WEPEFYEAM	B4001	R	916	924
**Y202**	GERVRQALL	B4001	R	179	187
**Y244**	AALTNNVAF	B4601	R	399	407
**Y246**	FAQDGNAAI	B4601, B5101	R	442	450
**Y275**	LPYPDPSRI	B5101, B5201	R	829	837
**Y276**	FAYTKRNVI	B5101, B5201	R	528	536

**Y306**	KSAGFPFNKW	B5801	R	500	509
**Y308**	MSYEDQDALF	B5801	R	519	528
**Y17**	LPNNTASWF	B0702, B3501, B5101	N	45	53
**Y18**	NPANNAAIV	B0702, B5101	N	150	158
**Y61**	LQLPQGTTL	B1301, B1302, B1501, B1502, B4001, B4801, B5201	N	159	167
**Y62**	KAYNVTQAF	B1301, B1302, B1501, B1502, B3501, B4403, B4601, B4801, B5101, B5201, B5801	N	266	274
**Y63**	MEVTPSGTW	B1301, B4001, B4403, B5801	N	322	330
**Y64**	RQKKQQTVTL	B1301, B1302, B1501, B4001, B4801	N	385	394
**Y65**	GDAALALLL	B1301, B4001, B4403	N	215	223
**Y104**	AQFAPSASA	B1302, B4001, B4801, B5201	N	305	313
**Y105**	GQQQQGQTV	B1302, B4001, B5201	N	238	246
**Y107**	SAFFGMSRI	B1302, B4601, B5101, B5201	N	312	320
**Y135**	FSKQLQQSM	B1501, B1502, B4601	N	403	411
**Y162**	ELIRQGTDY	B1502	N	290	298
**Y180**	LPAADLDDF	B3501	N	395	403
**Y196**	KKQQTVTLL	B4001, B4801	N	387	395
**Y222**	RQGTDYKHW	B4403	N	293	301
**Y224**	GKMKDLSPRW	B4403	N	99	108
**Y241**	NTASWFTAL	B4601	N	48	56
**Y242**	MAGNGGDAAL	B4601	N	210	219
**Y243**	LTYTGAIKL	B4601, B5201, B5801	N	331	339
**Y273**	LPYGANKDGI	B5101	N	121	130
**Y301**	GANKDGIIW	B5801	N	124	132
**Y303**	LSPRWYFYY	B5801	N	104	112
**Y43**	RPLLESELVI	B0702, B1302, B5101	M	131	140
**Y44**	RLFARTRSM	B0702, B1301, B1302, B1501, B1502, B4601, B4801	M	101	109
**Y47**	FARTRSMWSF	B0702, B1501, B1502, B3501, B4601	M	103	112
**Y49**	FAYANRNRF	B0702, B1501, B1502, B3501, B4601, B5101, B5201	M	37	45
**Y50**	WPVTLACFV	B0702, B3501, B5101	M	58	66
**Y90**	SELVIGAVI	B1301, B1302, B4001, B4403, B4801, B5201	M	136	144

**Y96**	FVLAAVYRI	B1301, B1302, B5101, B5201	M	65	73
**Y158**	VATSRTLSY	B1501, B1502, B3501, B4601, B5801	M	170	178
**Y176**	YANRNRFLY	B1502, B3501, B4601, B5801	M	39	47
**Y211**	LESELVIGAV	B4001, B4403	M	134	143
**Y215**	LEQWNLVIG	B4001	M	17	25
**Y216**	ADSNGTITV	B4001	M	2	10
**Y237**	EELKKLLEQW	B4403	M	11	20
**Y253**	IAMACLVGL	B4601, B4801	M	82	90
**Y297**	WSFNPETNI	B5201	M	110	118
**Y319**	LAAVYRINW	B5801	M	67	75
**Y320**	LVIGFLFLTW	B5801	M	22	31
**Y3**	YVYSRVKNL	B0702, B1502, B4601, B4801, B5101, B5201	E	57	65
**Y7**	LAILTALRL	B0702, B1301, B3501, B4001, B4601, B4801, B5101, B5201, B5801	E	31	39
**Y52**	LLFLAFVVF	B1301, B1501, B1502, B3501, B4001, B4403, B4601, B4801, B5201	E	18	26
**Y125**	LVKPSFYVY	B1501, B1502, B3501, B4403, B4601, B5801	E	51	59

Table S2: A total of 91 HLA-C restricted CD8⁺ T-cell epitope candidates were synthesized for further validation.

ID	Sequence	HLA-C allotypes binding to each epitope candidate as *in silico* predicted	Protein	Start	End
**M31**	YQPYRVVVL	C0102, C0401, C0602, C1402	S	505	513
**M33**	WTFGAGAAL	C0102, C0302, C0303, C0304, C0801, C1202, C1203, C1402, C1502	S	886	894
**M34**	KIYSKHTPI	C0102, C1402, C1502	S	202	210
**M35**	YLQPRTFLL	C0102, C0401, C0702, C0801, C1402	S	269	277
**M36**	MIAQYTSAL	C0102, C0302, C0303, C0304, C0401, C0801, C1202	S	869	877
**M37**	IANQFNSAI	C0102, C0302, C0303, C0304, C0801, C1202, C1203, C1502	S	923	931
**M38**	TVYDPLQPEL	C0102, C0303, C1402	S	1,136	1,145
**M40**	RVYSTGSNV	C0102, C1502	S	634	642
**M63**	FAMQMAYRF	C0302, C1202, C1203	S	898	906
**M64**	YSSANNCTF	C0302, C0401, C0701, C1202	S	160	168
**M66**	AALQIPFAM	C0302, C0303, C0304, C0801	S	892	900
**M68**	FVSNGTHWF	C0302, C0701, C1202	S	1,095	1,103
**M69**	QSAPHGVVF	C0302, C1202	S	1,054	1,062
**M82**	FVFLVLLPL	C0303, C0304	S	2	10
**M86**	SIIAYTMSL	C0304, C0801	S	691	699
**M102**	RLQSLQTYV	C0401	S	1000	1008
**M106**	VYDPLQPEL	C0401	S	1137	1145
**M124**	FRKSNLKPF	C0602, C0701, C0702	S	456	464
**M125**	KRFDNPVLPF	C0602, C0701, C0702	S	77	86
**M126**	YTNSFTRGV	C0602, C1203, C1502	S	28	36
**M127**	YSKHTPINL	C0602, C1203	S	204	212
**M128**	YHKNNKSWM	C0602, C0702	S	145	153
**M129**	NSFTRGVYY	C0602, C0701, C1203	S	30	38
**M131**	VVFLHVTYV	C0602, C1502	S	1,060	1,068
**M144**	VRFPNITNL	C0701, C0702	S	327	335
**M156**	YRVVVLSFEL	C0702	S	508	517
**M157**	AHFPREGVF	C0702	S	1087	1095

**M161**	ITDAVDCAL	C0801	S	285	293
**M180**	TFEYVSQPF	C1402	S	167	175
**M21**	AMYTPHTVL	C0102, C1402	R	923	931
**M22**	FAQDGNAAI	C0102, C0302, C0303, C0304, C0801, C1202, C1203, C1402	R	442	450
**M23**	FAYTKRNVI	C0102, C0302, C0303, C0304, C0602, C0701, C0801, C1202, C1203, C1402, C1502	R	528	536
**M24**	FVNEFYAYL	C0102, C0302, C0303, C0304, C0401, C0602, C0701, C0702, C0801, C1202, C1203, C1502	R	742	749
**M26**	YAYLRKHFSM	C0102, C0302, C0303, C0304, C0701, C1202, C1203, C1402	R	746	755
**M27**	LANECAQVL	C0102, C0302, C0303, C0304, C0602, C0701, C0801, C1202, C1203, C1502	R	655	663
**M29**	AVTANVNAL	C0102	R	699	707
**M30**	RAMPNMLRI	C0102, C0602, C0701, C1502	R	624	632
**M58**	LVYAADPAM	C0302, C0303, C0304	R	372	380
**M62**	MSYEDQDAL	C0302, C0303, C0304, C0801	R	519	527
**M79**	IAATRGATV	C0303, C0304, C1203, C1502	R	579	587
**M94**	FFKEGSSVEL	C0401, C0702, C1402	R	428	437
**M95**	YYSLLMPIL	C0401	R	237	245
**M96**	FVDGVPFVV	C0401, C0801	R	334	342
**M98**	YYRYNLPTM	C0401, C0602, C0701, C0702, C1402	R	455	463
**M99**	LYYQNNVFM	C0401, C0702, C1402	R	786	794
**M119**	SAKNRARTV	C0602, C1203	R	549	557
**M120**	YVRNLQHRL	C0602	R	719	727
**M122**	YFKYWDQTY	C0602, C0702, C1402	R	286	294
**M151**	YFVVKRHTF	C0702	R	69	77
**M152**	YHPNCVNCL	C0702	R	294	302
**M158**	YAADPAMHAA	C0801	R	374	383
**M159**	LMIERFVSL	C0801, C1202	R	854	862
**M11**	KAYNVTQAF	C0102, C0302, C0303, C0304, C0602, C0701, C0702, C0801, C1202, C1203, C1402, C1502	N	266	274
**M12**	LLLDRLNQL	C0102, C0303, C0304, C0401, C0702, C0801, C1202	N	222	230
**M13**	LQLPQGTTL	C0102, C0302, C0303, C0304, C0401, C0801, C1202	N	159	167
**M14**	FSKQLQQSM	C0102, C0302, C0303, C0304, C0602, C0701, C0702, C0801, C1202, C1203, C1402, C1502	N	403	411
**M15**	LTYTGAIKL	C0102, C0302, C0303, C0304, C0602, C0701, C0801, C1202, C1203, C1402, C1502	N	331	339

**M16**	FAPSASAFF	C0102, C0302, C0303, C0401, C0602, C0801, C1202, C1203, C1402	N	307	315
**M18**	VLQLPQGTTL	C0102	N	158	167
**M19**	FGMSRIGMEV	C0102, C0602, C1203	N	315	324
**M56**	NTASWFTAL	C0302, C0303, C0304, C0801, C1202, C1502	N	48	56
**M77**	SAFFGMSRI	C0303, C0304, C0602, C0801, C1202, C1203, C1502	N	312	320
**M88**	NFKDQVILL	C0401	N	345	353
**M89**	FFGMSRIGM	C0401, C0702, C1402	N	314	322
**M90**	KHWPQIAQF	C0401, C0701, C0702	N	299	307
**M115**	LKFPRGQGV	C0602, C0701, C1203	N	64	72
**M117**	TRNPANNAAI	C0602, C0701	N	148	157
**M136**	KHIDAYKTF	C0701, C0702	N	355	363
**M150**	FYYLGTGPEA	C0702, C1402	N	110	119
**M186**	RTATKAYNV	C1502	N	262	270
**M41**	RLFARTRSM	C0102, C0302, C0303, C0304, C0602, C0701, C0702, C1202, C1203, C1402	M	101	109
**M43**	FAYANRNRF	C0102, C0302, C0303, C0304, C0401, C0602, C0701, C0702, C0801, C1202, C1203, C1402, C1502	M	37	45
**M44**	IAMACLVGL	C0102, C0302, C0303, C0304, C0801, C1202, C1203, C1402, C1502	M	82	90
**M45**	FLFLTWICL	C0102	M	26	34
**M47**	SFNPETNIL	C0102, C0401, C0702, C1402	M	111	119
**M48**	MADSNGTITV	C0102, C0303, C0304, C0401, C0801, C1502	M	1	10
**M49**	ITVATSRTL	C0102, C0303, C0304, C1502	M	168	176
**M71**	FARTRSMWSF	C0302, C1202, C1203	M	103	112
**M72**	VATSRTLSY	C0302, C0602, C0701, C1202, C1203, C1402	M	170	178
**M73**	YANRNRFLYI	C0302, C0303, C0304, C0401, C0602, C0801, C1203, C1502	M	39	48
**M74**	FAAYSRYRI	C0302, C0303, C0304, C0801, C1502	M	193	201
**M84**	IAIAMACLV	C0303, C0304, C0801, C1203, C1502	M	80	88
**M107**	YFIASFRLF	C0401, C0702, C1402	M	95	103
**M111**	SSSDNIALL	C0401, C0602, C0701, C0801, C1202	M	212	220
**M133**	YSRYRIGNY	C0602, C1202, C1203 C0702	M	196	204
**M134**	YRINWITGGI	C0602	M	71	80
**M1**	YVYSRVKNL	C0102, C0302, C0303, C0304, C0401, C0602, C0701, C0702, C0801, C1202, C1203, C1402, C1502	E	57	65

**M7**	IVNSVLLFL	C0102, C0302, C0303, C0304, C0401, C0602, C0701, C0702, C0801, C1202, C1203, C1502	E	13	21
**M51**	LTALRLCAY	C0302, C0701, C1202, C1203, C1402	E	34	42
**M76**	FLAFVVFLL	C0303, C0304, C0401, C0602, C0701, C0702, C0801, C1202	E	20	28
**M3**	VTLAILTAL	C0102, C0302, C0303, C0304, C0401, C0801, C1202, C1203, C1402, C1502	E	29	37

Table S3: Binding affinities of each HLA-B allotype with corresponding VEPs as detected by competitive peptide binding assays using transfected HMy2.CIR cell lines expressing indicated HLA-B allotype.

**HLA-B4601**		**HLA-B4001**		**HLA-B5801**		**HLA-B1502**		**HLA-B5101**		**HLA-B1301**		**HLA-B1302**		**HLA-B1501**		**HLA-B3501**		**HLA-B4403**		HLA-B5201		HLA-B4801		HLA-B 0702	
Y243	High	Y104	High	Y175	High	Y174	High	Y96	High	Y65	High	Y104	High	Y297	High	Y17	High	Y230	High	Y313	Inter	Y224	Inter	Y308	High
Y231	High	Y215	Inter	Y142	High	Y47	High	Y276	Inter	Y276	High	Y306	High	Y158	High	Y61	High	Y3	High	Y308	Low	Y246	Low	Y189	High
Y28	High	Y216	Inter	Y247	High	Y175	Inter	Y61	Inter	Y81	High	Y151	High	Y125	High	Y199	High	Y118	Inter	Y303	Low	Y303	Low	Y301	Inter
Y189	High	Y71	Inter	Y301	High	Y158	Inter	Y313	Low	Y96	High	Y77	High	Y146	High	Y49	High	Y253	Inter	Y301	No	Y198	Low	Y165	Inter
Y253	High	Y211	Inter	Y303	High	Y275	Inter	Y71	Low	Y43	Inter	Y118	High	Y28	High	Y82	High	Y62	Inter			Y243	No	Y135	Inter
Y162	High	Y89	Inter	Y172	High	Y273	Inter	Y301	Low	Y143	Inter	Y89	High	Y135	High	Y151	High	Y77	Low			Y282	No	Y164	Low
Y196	High	Y297	Low	Y65	Inter	Y146	Inter	Y303	Low	Y104	Inter	Y199	Inter	Y172	High	Y47	High	Y89	Low			Y306	No	Y167	Low
Y135	High	Y306	Low	Y61	Inter	Y164	Inter	Y198	Low	Y215	Inter	Y118	Inter	Y65	Inter	Y62	High	Y167	Low			Y200	No	Y202	Low
Y215	Inter	Y230	Low	Y199	Inter	Y162	Inter	Y308	No	Y231	Inter	Y247	Inter	Y143	Inter	Y165	High	Y172	Low			Y142	No	Y211	No
Y308	Inter	Y308	Low	Y230	Inter	Y167	Inter	Y88	No	Y189	Inter	Y253	Inter	Y18	Inter	Y158	Inter	Y18	No			Y125	No	Y303	No
Y77	Inter	Y231	Low	Y49	Inter	Y165	Inter	Y82	No	Y253	Inter	Y119	Inter	Y222	Inter	Y88	Inter	Y77	No			Y301	No	Y180	No
Y247	Inter	Y142	Low	Y82	Inter	Y172	Inter	Y28	No	Y165	Inter	Y303	Inter	Y249	Inter	Y7	Inter							Y196	No
Y301	Inter	Y62	Low	Y216	Inter	Y276	Low	Y172	No	Y175	Low	Y196	Inter	Y281	Inter	Y71	Inter							Y62	No
Y167	Inter	Y165	Low	Y77	Inter	Y243	Low			Y249	Low	Y96	Inter	Y275	Inter	Y28	Inter							Y172	No
Y202	Inter	Y172	Low	Y125	Inter	Y283	Low			Y281	Low	Y198	Inter	Y282	Inter	Y189	Inter								
Y62	Inter	Y18	No	Y7	Inter	Y231	Low			Y275	Low	Y222	Low	Y283	Inter	Y167	Inter								
Y165	Inter	Y174	No	Y164	Inter	Y142	Low			Y282	Low	Y249	Low	Y142	Inter	Y96	Inter								
Y18	Low	Y164	No	Y162	Inter	Y125	Low			Y200	Low	Y281	Low	Y174	Inter	Y172	Inter								
Y81	Low			Y62	Inter	Y246	Low			Y283	Low	Y17	Low	Y71	Inter	Y65	Low								
Y224	Low			Y135	Inter	Y28	Low			Y216	Low	Y49	Low	Y164	Inter	Y143	Low								
Y199	Low			Y158	Low	Y301	Low			Y28	Low	Y250	Low	Y211	Inter	Y224	Low								
Y230	Low			Y275	Low	Y202	Low			Y247	Low	Y211	Low	Y180	Inter	Y243	Low								
Y308	Low			Y200	Low	Y180	Low			Y119	Low	Y3	Low	Y3	Inter	Y200	Low								
Y216	Low			Y215	Low	Y281	No			Y167	Low	Y62	Low	Y62	Inter	Y215	Low								
Y174	Low			Y273	Low	Y282	No			Y180	Low	Y172	Low	Y175	Low	Y230	Low								
Y47	Low			Y88	Low	Y230	No			Y196	Low	Y200	No	Y276	Low	Y250	Low								
Y118	Low			Y174	Low	Y82	No			Y3	Low	Y216	No	Y17	Low	Y118	Low								
Y211	Low			Y7	Low	Y250	No			Y297	No	Y231	No	Y81	Low	Y246	Low								
Y89	Low			Y246	Low	Y118	No			Y224	No	Y125	No	Y306	Low	Y164	Low								
Y303	Low			Y189	Low	Y247	No			Y243	No	Y47	No	Y200	Low	Y211	Low								
Y180	Low			Y253	Low	Y189	No			Y77	No	Y71	No	Y230	Low	Y119	Low								
Y96	Low			Y89	Low	Y211	No			Y246	No	Y246	No	Y273	Low	Y162	Low								
Y198	Low			Y119	Low	Y253	No			Y164	No	Y301	No	Y49	Low	Y303	Low								
Y172	Low			Y167	Low	Y198	No			Y211	No	Y89	No	Y88	Low	Y196	Low								

Y61	No			Y202	Low					Y167	No	Y162	No	Y82	Low	Y3	Low								
Y273	No			Y143	No					Y202	No	Y202	No	Y231	Low	Y135	Low								
Y7	No			Y17	No					Y3	No	Y303	No	Y247	Low	Y175	No								
Y164	No			Y151	No					Y135	No			Y301	Low	Y276	No								
Y3	No			Y47	No					Y172	No			Y253	Low	Y216	No								
				Y71	No									Y89	Low	Y142	No								
				Y118	No									Y162	Low	Y174	No								
				Y28	No									Y167	Low	Y77	No								
				Y211	No									Y202	Low	Y146	No								
				Y180	No									Y196	Low	Y247	No								
				Y196	No									Y96	Low	Y253	No								
				Y96	No									Y165	Low	Y89	No								
				Y165	No									Y198	Low	Y202	No								
				Y198	No									Y224	No	Y198	No								
														Y243	No										
														Y151	No										
														Y250	No										
														Y246	No										
														Y189	No										
														Y119	No										

Table S4: Binding affinities of each HLA-C allotype with corresponding VEPs as detected by competitive peptide binding assays using transfected HMy2.CIR cell lines expressing indicated HLA-C allotype.

HLA-C0102		HLA-C0602		HLA-C0702		HLA-C0801		HLA-C0304		HLA-C0302		HLA-C0303		HLA-C0401		HLA-C1402		HLA-C1202		HLA-C1502		HLA-C 0701	
M3	High	M51	High	M159	High	M72	High	M76	High	M16	High	M23	High	M3	High	M107	High	M156	Low	M64	High	M43	High
M34	High	M73	High	M117	High	M96	High	M13	High	M71	High	M37	High	M111	High	M95	Inter	M131	Low	M68	Inter	M71	Inter
M37	High	M117	High	M47	High	M15	High	M66	High	M22	High	M14	High	M16	Inter	M96	Inter					M68	Low
M47	High	M15	High	M35	High	M22	High	M26	Inter	M63	High	M159	Inter	M34	Inter	M35	Inter					M44	Low
M7	High	M30	High	M3	Inter	M41	Inter	M48	Inter	M44	High	M107	Inter	M86	Inter	M86	Low					M22	No
M44	High	M19	High	M76	Inter	M56	Inter	M131	Inter	M58	High	M63	Inter	M13	Inter	M117	Low						
M51	Inter	M11	High	M122	Inter	M23	Low	M152	Inter	M14	High	M58	Inter	M7	Inter	M156	Low						
M159	Inter	M125	Inter	M13	Inter	M107	Low	M22	Inter	M33	Inter	M96	Low	M152	Inter	M13	Low						
M23	Inter	M102	Inter	M43	Inter	M37	Low	M14	Inter	M73	Inter	M22	Low	M72	Low	M69	Low						
M16	Inter	M111	Inter	M71	Inter	M68	Low	M33	Low	M26	Inter	M64	Low	M43	Low	M76	No						
M86	Inter	M43	Inter	M11	Inter	M30	Low	M34	Low	M76	Inter	M44	Low	M30	Low	M111	No						
M95	Inter	M7	Inter	M14	Inter	M69	Low	M156	Low	M64	Inter	M156	No	M69	Low	M7	No						
M156	Inter	M66	Inter	M102	Low	M152	Low	M7	Low	M34	Low			M71	Low								
M30	Inter	M127	Low	M72	Low	M71	Low	M72	No	M122	Low			M11	Low								
M18	Inter	M157	Low	M41	Low	M64	Low	M122	No	M15	Low			M58	Low								
M131	Inter	M180	Low	M26	Low	M44	Low	M18	No	M47	Low			M23	No								
M11	Inter	M3	Low	M95	Low	M14	Low	M35	No	M7	Low			M37	No								
M73	Low	M16	Low	M107	Low	M18	No	M44	No	M11	Low			M63	No								
M56	Low	M26	Low	M15	Low	M19	No			M129	No												
M111	Low	M95	Low	M30	Low	M63	No			M72	No												
M117	Low	M122	Low	M7	Low	M58	No			M86	No												
M122	Low	M48	Low	M19	Low					M35	No												
M43	Low	M68	Low	M131	Low					M131	No												
M48	Low	M71	Low	M152	Low																		
M69	Low	M161	No	M63	Low																		
M19	Low	M56	No	M58	Low																		
M35	Low	M107	No	M127	No																		
M152	Low	M37	No	M16	No																		
M22	Low	M69	No	M96	No																		
M64	Low	M18	No	M111	No																		
M63	Low	M152	No	M68	No																		
M58	Low	M22	No	M64	No																		
M14	Low	M63	No	M44	No																		
M129	No	M44	No																				

M41	No	M58	No																				
M26	No	M14	No																				
M96	No																						
M107	No																						
M68	No																						
M66	No																						
